# RNA helicase MOV10 suppresses fear memory and dendritic arborization and regulates microtubule dynamics in hippocampal neurons

**DOI:** 10.1186/s12915-025-02138-6

**Published:** 2025-02-06

**Authors:** Temirlan Shilikbay, Aatiqa Nawaz, Megan Doon, Stephanie Ceman

**Affiliations:** 1 Cell and Developmental Biology, University of Illinois Urbana-Champaign, Urbana, USA; 2 Molecular and Cellular Biology, University of Illinois Urbana-Champaign, Urbana, USA; 3 Neuroscience Program, University of Illinois Urbana-Champaign, Urbana, IL USA

**Keywords:** MOV10, RNA helicase, AGO2, Hippocampal neurons, Cytoskeleton, NUMA1, Dendrites, Translation regulation

## Abstract

**Background:**

RNA helicase MOV10 is highly expressed in postnatal brain and associates with FMRP and AGO2, suggesting a role in translation regulation in learning and memory.

**Results:**

We generated a brain-specific knockout mouse (*Mov10* Deletion) with greatly reduced MOV10 expression in cortex and hippocampus. Behavior testing revealed enhanced fear memory, similar to that observed in a mouse with reduced brain microRNA production, supporting MOV10’s reported role as an AGO2 cofactor. Cultured hippocampal neurons have elongated distal dendrites, a reported feature of augmin/HAUS over-expression in *Drosophila* da sensory neurons. In mitotic spindle formation, HAUS is antagonized by the microtubule bundling protein NUMA1. *Numa1* mRNA is a MOV10 CLIP target and is among the genes significantly decreased in *Mov10* Deletion hippocampus. Restoration of NUMA1 expression and knockdown of HAUS rescued phenotypes of the *Mov10* Deletion hippocampal neurons.

**Conclusions:**

This is the first evidence of translation regulation of NUMA1 by MOV10 as a control point in dendritogenesis.

**Supplementary Information:**

The online version contains supplementary material available at 10.1186/s12915-025-02138-6.

## Background

The formation of dendrites occurs through the translation of messenger RNAs (mRNAs) and the mobilization of cytoskeletal proteins in response to cell intrinsic and extrinsic cues [1, 2]. RNA-Binding Proteins (RBPs) regulate the accessibility of the mRNA to other proteins, including Argonaute 2 (AGO2), which is associated with microRNAs (miRNAs). The AGO2-miRNA complex must be able to access the miRNA recognition element (MRE) in the 3’ untranslated region (UTR) of the target mRNAs. As AGO2 does not have unwinding activity, there is a need for RNA helicases like MOV10 to unwind secondary structures to reveal MREs. Accordingly, MOV10 was identified as a functional cofactor of AGO2 because it was required for miRNA-mediated silencing of an EGFP reporter [3]. This observation predicts that in the absence of MOV10, AGO2 target mRNAs would be over-expressed,however, we found that loss of MOV10 does not consistently lead to increased expression of AGO2 target mRNAs [4]. In fact, MOV10 protects a subset of mRNAs from AGO2-mediated silencing based on cis features in the mRNAs like G-quadruplexes as well as the presence of other RBPs like FMRP [4, 5]. Thus, a combination of the sequences in the mRNA and associated RBPs like FMRP and MOV10 control the access of AGO2 to its MREs.

In cultured hippocampal neurons, MOV10 is distributed throughout the cell body and dendrites, often in puncta [6]. In addition, MOV10 is one of the most enriched proteins in the neurites of induced neurons [7], suggesting an important role in extension and/or branching. MOV10 is also significantly elevated in postnatal brain from P0-P14 when dendrites are forming [8].

To establish a role for MOV10 in neuronal development and function, it was necessary to create a brain-specific knockout mouse because complete loss of MOV10 in both *Xenopus laevis* and mouse results in early embryonic lethality [8, 9]. We show here that loss of MOV10 expression in neocortex and hippocampus leads to increased cortical thickness, increased fear memory, and increased dendritic length of cultured hippocampal neurons but not necessarily increased branch points. To determine the point during dendritic development where MOV10 acts, we examined dendritic growth cones and observed impaired microtubule comet formation and traveling rate. To identify the MOV10-dependent mRNAs that might be participating in dendritogenesis, we performed RNA-sequencing and found that a group of significantly changed mRNAs encode cytoskeletal proteins, including microtubule binding proteins. Querying this list for directly bound mRNAs, i.e., MOV10 UV-Cross Linking-Immunoprecipitation (CLIP) targets, we identified the Nuclear Mitotic Apparatus binding protein 1 mRNA (*Numa1*). An earlier study identified NUMA1 in dendrites but its function there was unknown [10]. NUMA1 has been extensively studied as a microtubule binding protein that participates in spindle formation in dividing cells. In this role, NUMA1 has an antagonistic relationship with Human Augmin complex (HAUS) [11], which also participates in spindle formation. Relevant to the current work, unregulated HAUS in *Drosophila* sensory da neurons leads to increased dendritic arborization [12], a feature we observe in the *Mov10* Deletion neurons. To verify the role for NUMA1 and HAUS in mammalian dendrite development, we over-expressed NUMA1 and knocked down the sixth subunit of HAUS in MOV10 depleted hippocampal neurons and rescued microtubule comet formation in growth cones and reduced the increased dendritic arborization of *Mov10* Deletion neurons. Our work suggests that NUMA1, which usually functions in spindle formation in dividing cells has been co-opted for dendritogenesis in post-mitotic neurons. Importantly, we have identified a novel role for MOV10 in the regulated expression of proteins that participate in microtubule polymerization and ultimately dendritic arborization, which is required for normal memory.

## Results

### Brain-specific MOV10 knockout mouse has enhanced fear memory

**Fig. 1:**
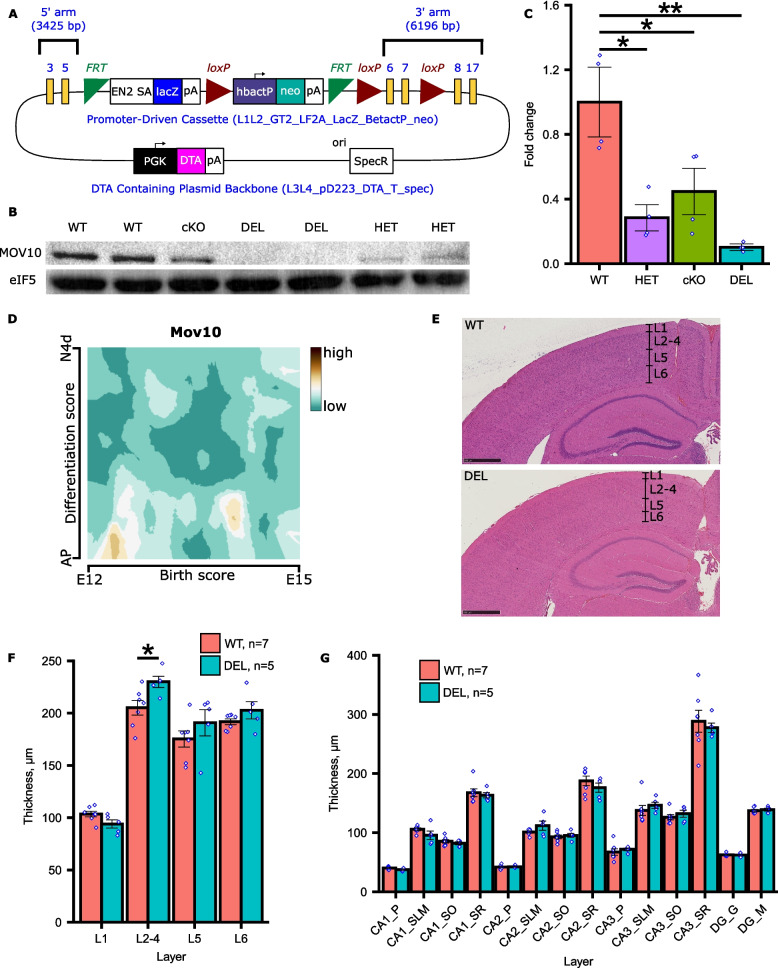
Creation and characterization of the *Mov10* Deletion mouse. **A** Targeting construct from EUCOMM used to create the *Mov10*^fl/fl^ mouse. **B** Hippocampal lysates from P2 mice of the indicated genotypes (WT-C57BL/6; cKO-*Mov10* conditional knockout; DEL-*Mov10* Deletion; HET-*Mov10* Heterozygote) were immunoblotted for MOV10 and eIF5 (loading control). Original uncropped blots are in Additional File 11. **C** Fold-change of normalized MOV10 expression in (**B**). *P*-values were calculated using two-sided unpaired Welch's t-test. **D** *Mov10* expression in apical progenitor (AP) cells (Y axis-Differentiation score) between embryonic days (**E**) 12–15 (X-axis) [18]. **E** Coronal sections of 12-week-old WT and *Mov10* Deletion (DEL) cortices stained with H&E. Borders between adjacent cortical layers (L1-6) were identified based on differences in cytoarchitecture [19]. Scale bar = 500 μm. **F** Thickness (mm) of cortical layers 1, 2–4 (combined), 5, and 6 in adult WT and *Mov10* Deletion (DEL) mice. *P*-values were calculated using two-sided unpaired Student’s t-test. **G** Thickness (mm) of hippocampal layers in adult WT and *Mov10* Deletion (DEL) mice. P – pyramidal layer, SLM – stratum lacunosum moleculare, SO – stratum oriens, SR – stratum radiatum, G – granular layer, M – molecular layer. Data are shown as mean ± SEM. *p-value < 0.05. **p-value < 0.01, n is the number of mice of the genotype indicated, both sexes from *N* > 2 litters

Our earlier work in *X. laevis* tadpoles suggested that MOV10 participated in some aspects of brain development, including the diencephalon [9]. To study the function of MOV10 in mammalian cortex and hippocampus, we created a *Mov10* conditional knockout (*Mov10* cKO) mouse by obtaining a targeting vector with LoxP sequences flanking exons 6 and 7 of the *Mov10* gene [13] (Fig. 1A). This vector was introduced into C57BL/6 embryonic stem cells and screened for homologous insertion into the *Mov10* locus to create the *Mov10*^*flox/flox*^ line (details are in the Methods and Supplemental Information). To obtain the *Mov10* cKO mouse, the floxed mouse was crossed to an *Emx1*^*Cre*^ mouse, leading to depletion of MOV10 in excitatory neurons of the neocortex and hippocampus [14–16]. As *Emx1* has been reported to be expressed in 88% of neurons in those structures, we expected an 88% reduction in MOV10 levels. However, analysis of postantal day 2 (P2) hippocampal lysates revealed only a 50% reduction in MOV10 expression (Fig. 1B and C). A possible explanation is that the chromatin is partially inaccessible, which has been described for other loci [17]. Because we had already determined that a 50% reduction of MOV10 did not result in impaired learning and memory [8], we achieved a greater reduction of MOV10 by crossing the *Mov10* cKO mouse with our previously described *Mov10* heterozygous mutant (*Mov10*^+/-^) [8] and named the resulting mouse *Mov10* Deletion. The MOV10 levels were now reduced by 90% in the *Mov10* Deletion mouse (Fig. 1B and C), which we proceeded to use in our experiments.

We began by examining the gross structure of the cortex because our previous work in tadpoles suggested a role for MOV10 in the localization of neuronal precursor cells and mature neurons around the ventricle [9]. In addition, MOV10 was among the loci identified in genome wide association studies as correlating with regional brain volumes [20–22]. For cortical development, neurons are sequentially born and differentiate from apical progenitor cells (APCs) located in the ventricular zone. We examined *Mov10* expression in murine APCs [18] and found that it was elevated at embryonic day 12 (E12) and at intermediate levels around E14 (Fig. 1D), which correspond to the development of cortical layers 6, 5, and 4, respectively. When we measured the cortical layers in adult WT and *Mov10* Deletion mice, we found no difference in the size of layers 6, 5 and 1; however, there was an increased thickness of layers 2–4 (Fig. 1E and F) with no increase in cell density (Additional File 1: Fig. S1A). Thus, loss of MOV10 leads to an increase in both the number of cells and neuropil in layers 2–4. We also examined the hippocampus but observed no significant differences in cell density (Fig. 1G, S1B). Thus, MOV10 expression in apical progenitor cells may participate in migration, apoptosis and/or neuropil production in cortical layers 2–4 but has no effect on the overall structure of the hippocampus.

**Fig. 2:**
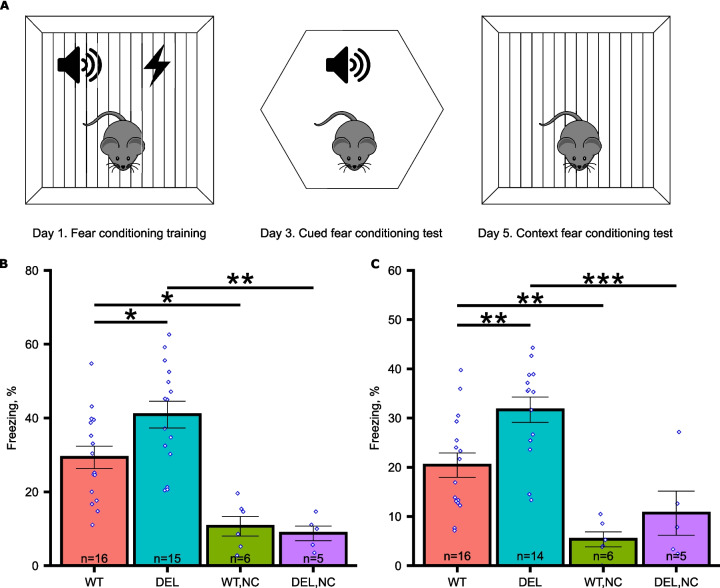
*Mov10* Deletion mouse shows enhanced fear memory. **A** Schematic of the tone-shock training and the fear memory tests. **B** Cued fear conditioning memory test with percent of time freezing in a new context (Y axis) of WT and *Mov10* Deletion (DEL) mice. NC-non-shock control mice that did not receive foot shock during training. “n” = the number of mice of the genotype indicated, both sexes were used from *N* > 3 litters. **C** Context fear conditioning test with percent of time freezing during re-exposure to the training context two days after training (Y axis). Data are shown as mean ± SEM. *P*-values were calculated using Mixed Effect ANOVA with sex, age, and batch as random effects and Tukey HSD test. *p-value < 0.05. **p-value < 0.01, ***p-value < 0.001

We next evaluated the role of MOV10 in behavior by examining whether normal MOV10 levels were required for tasks of learning and memory [23]. Using Y-maze and T-maze, we examined the number of prior arm visits—as a proxy for memory—by measuring the rate of alteration between the arms. Although it was higher for the *Mov10* Deletion, suggesting enhanced memory of prior arm visits, it was not significantly different than WT (Additional File 1: Fig. S1C and D). Only in the tests of fear memory did we find a significant difference between the WT and *Mov10* Deletion mouse performance with the *Mov10* Deletion mouse having enhanced memory (Fig. 2, Additional File 2: Fig. S2). Specifically, the *Mov10* Deletion mouse showed significantly more freezing behavior upon hearing the tone in a new context (Fig. 2B). Further, when the *Mov10* Deletion mouse was placed back in the original training arena, it showed increased freezing compared to WT (Fig. 2C), indicating enhanced contextual fear memory, which is a function of the hippocampus. To rule out the possibility that the *Mov10* Deletion mice have an increased sensitivity to pain, we examined the initial response to shock by both *Mov10* Deletion and WT mice during training on day 1 and found no significant difference between the genotypes in response to shock (Additional File 2: Fig. S2C and D), suggesting that the increased freezing on days 2 and 3 are memory phenotypes. We appreciate that the absolute value of percent freezing in both cued and contextual fear conditioning experiments was lower than expected [24], which could be explained by our use of a stress-reducing handling tunnel compared to handling by the tail [25]. In other tests of behavior, there was no significant difference between genotypes in the novel object recognition (Additional File 1: Fig. S1E) nor in tests of hyperactivity or anxiety (Additional File 1: Fig. S1F, G, and H), the last of which was unexpected because the *Mov10*^±^ mouse had increased anxiety [8]. Perhaps having half as much MOV10 throughout the brain led to an anxious phenotype, which is now corrected by the 90% loss in the excitatory neurons of neocortex and hippocampus. In conclusion, loss of MOV10 leads to enhanced fear memory, pointing to a new role for MOV10 in fear memory suppression.

### Cultured hippocampal neurons from the Mov10 deletion mouse have increased dendritic arborization

**Fig. 3:**
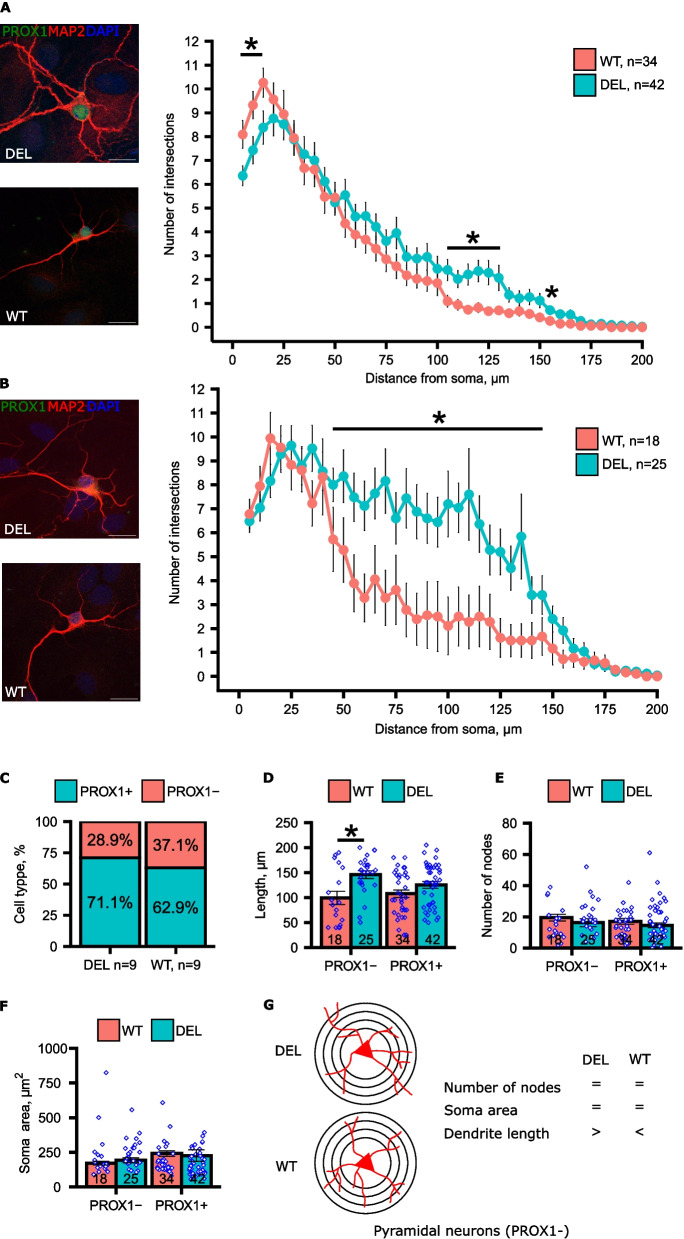
Increased distal dendritic arborization in *Mov10* Deletion hippocampal neurons. **A**, **B** Representative images of confocal z-stacks of MAP2 (red) and PROX1 (green)-stained WT and *Mov10* Deletion (DEL) DIV14 neurons analyzed using Sholl analysis: the number of intersections (Y axis) and the distance from the soma (mm) (X axis). **A** PROX1 + granule cells **B** PROX1.^−^ pyramidal neurons. Scale bars represent 20 μm. “n” = number of neurons cultured from *N* = 3 litters cultured separately. **C** Percentage of PROX1 + granule cells and PROX1- pyramidal neurons in the DIV14 *Mov10* Deletion and WT primary hippocampal cultures. **D** Length (μm) of the longest dendrites of the DIV14 WT and *Mov10* Deletion hippocampal neurons. **E** Number of nodes in the dendritic arbors of the DIV14 WT and *Mov10* Deletion hippocampal neurons. **F** Soma area of the DIV14 WT and *Mov10* Deletion hippocampal neurons. **G** Schematic representation of the dendritic phenotype observed in *Mov10* Deletion hippocampal pyramidal neurons. *P*-values were calculated using Mann–Whitney U test. Data are shown as mean ± SEM. *p-value < 0.05

Cued fear conditioning and context fear conditioning are controlled in large part by the hippocampus [26]. To examine the effect of MOV10 depletion on hippocampal neuron morphology, we performed Sholl analysis on day in vitro (DIV) 14 hippocampal neurons (Fig. 3). We found that the *Mov10* Deletion granule cells (PROX1 +) have significantly decreased proximal arborization at 5–15 µm from the soma but significantly increased distal arborization at 105–130 µm and 155 µm from the soma compared to WT (Fig. 3A, S3A). In contrast, the *Mov10* Deletion pyramidal neurons were not significantly different than WT proximal to the soma; however, had significantly increased distal arborization at 40–145 µm from the soma compared to WT (Fig. 3B). Thus, in both hippocampal neuronal subtypes–the distribution of which was not different between genotypes (Fig. 3C)–the loss of MOV10 led to increased distal arborization. This observation suggests that MOV10 participates in regulated dendritic branching.

To identify the dendritic feature that participates in the increased arborization measured by Sholl, we examined the dendrite length and the number of branch points (nodes) and found that the pyramidal neurons (PROX-) had significantly increased dendrite length compared to WT (Fig. 3D) but no change in the number of nodes (Fig. 3E). This result suggests that the increased arborization is due to increased dendrite length and not branching when MOV10 is reduced by 90%. In earlier work, the *Mov10*^+/-^ hippocampal cultures also showed decreased proximal arborization but did not show the increased distal arborization observed here, suggesting that the concentration of MOV10 is critical in determining the final shape of the neuron. The *Mov10*^+/-^ neuron also had an increased soma size [27], which we also did not observe (Fig. 3F). Our results suggest that the near complete loss of MOV10 in hippocampal neurons perturbs the regulation of dendrite elongation in hippocampal neurons, particularly in the pyramidal neurons (Fig. 3G).

### Dendritic growth cones require MOV10 for microtubule comet initiation and polymerization

**Fig. 4:**
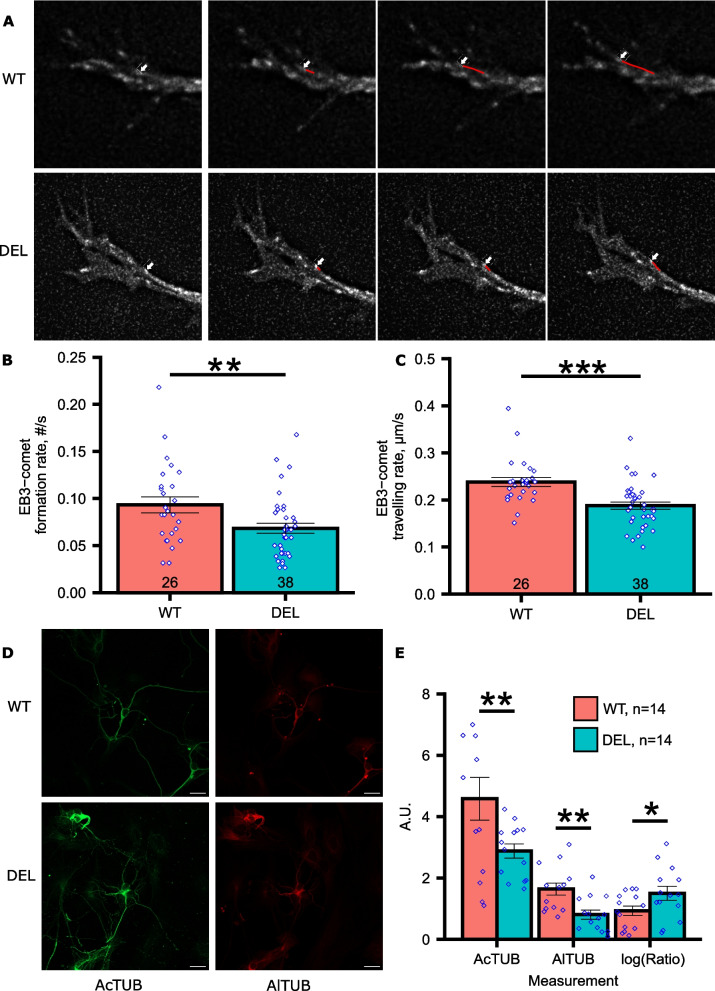
Dendritic growth cones of *Mov10* Deletion hippocampal neurons have less polymerizing plus-end microtubules but higher acetylation. **A** Representative images of the EB3-comets in the dendritic growth cones of DIV2-4 WT and *Mov10* Deletion (DEL) hippocampal neurons over time (seconds). A white arrow identifies a single EB3-comet whose trajectory over time is indicated by a red line. **B** Rate of EB3 comet formation is the number of comets/second (Y axis). **C** Traveling rate of EB3 comets is the distance in μm/second (Y axis). Number of neurons imaged from each genotype is indicated in the bars. *P*-values were calculated using Mann–Whitney U test. **D** Representative images of DIV7 WT and *Mov10* Deletion hippocampal neurons stained for acetylated tubulin (AcTUB) and for alpha tubulin (AlTUB). Scale bar = 20 μm. **E** Fluorescent intensity of AcTUB and AlTUB and ratio of AcTUB/AlTUB in WT and *Mov10* Deletion DIV7 hippocampal neurons. Data are shown as mean ± SEM. *P*-values were calculated using two-sided unpaired Student’s T-test. *p-value < 0.05, **p-value < 0.01, ***p-value < 0.001. “n” = number of neurons in the genotypes indicated from *N* = 3 litters cultured separately

To obtain insight into the cause of the dendritic phenotype, we examined the cytoskeletal dynamics in dendritic growth cones by expressing a dual reporter [EB3-mCherry/Lifeact-GFP [28]] that labeled the polymerizing plus-end microtubules (EB3-mCherry, red) and actin (Lifeact-GFP, green). We then performed live imaging of DIV2 neurons because this timeframe occurs after axon specification [29], allowing us to focus on the developing dendrites. We observed significantly reduced rates of nucleation of new EB3-comets and a reduced polymerization rate in the absence of MOV10 (Fig. 4A-C), suggesting that MOV10 is required for microtubule comet formation and growth. We also observed no change in actin-regulated filopodia retraction nor in the average distance traveled by EB3-comets (Additional File 3: Fig. S3B and C). Thus, MOV10 is required for microtubule initiation and traveling rate in dendritic growth cones.

Although microtubule dynamics were reduced in the absence of MOV10, we hypothesized that increased microtubule stability may participate in the increased dendrite length. Because acetylation marks stable microtubules [30], we stained hippocampal extracts for acetylated tubulin but did not observe a difference (Additional File 3: Fig. S3D); however, staining of neurons revealed that the ratio of acetylated-tubulin to alpha-tubulin was significantly greater in *Mov10* Deletion neurons, despite there being less tubulin overall in the absence of MOV10 (Fig. 4D and E, Additional File 3: Fig. S3E). As alpha tubulin is the primary component of microtubules, its reduction must mean less microtubules. However, while there are less microtubules in the *Mov10* Deletion neurons, the microtubules that are present are more stable, i.e., acetylated, than the microtubules in WT neurons. We conclude that MOV10 facilitates microtubule initiation and polymerization in the dendritic growth cones, regulates tubulin levels, and normalizes microtubule stability. As MOV10 is a potent regulator of mRNA stability [4], it was imperative to identify the significantly changed mRNAs that encode proteins involved in microtubule regulation.

### Differentially expressed mRNAs in the Mov10 Deletion hippocampus encode cytoskeletal proteins

**Fig. 5:**
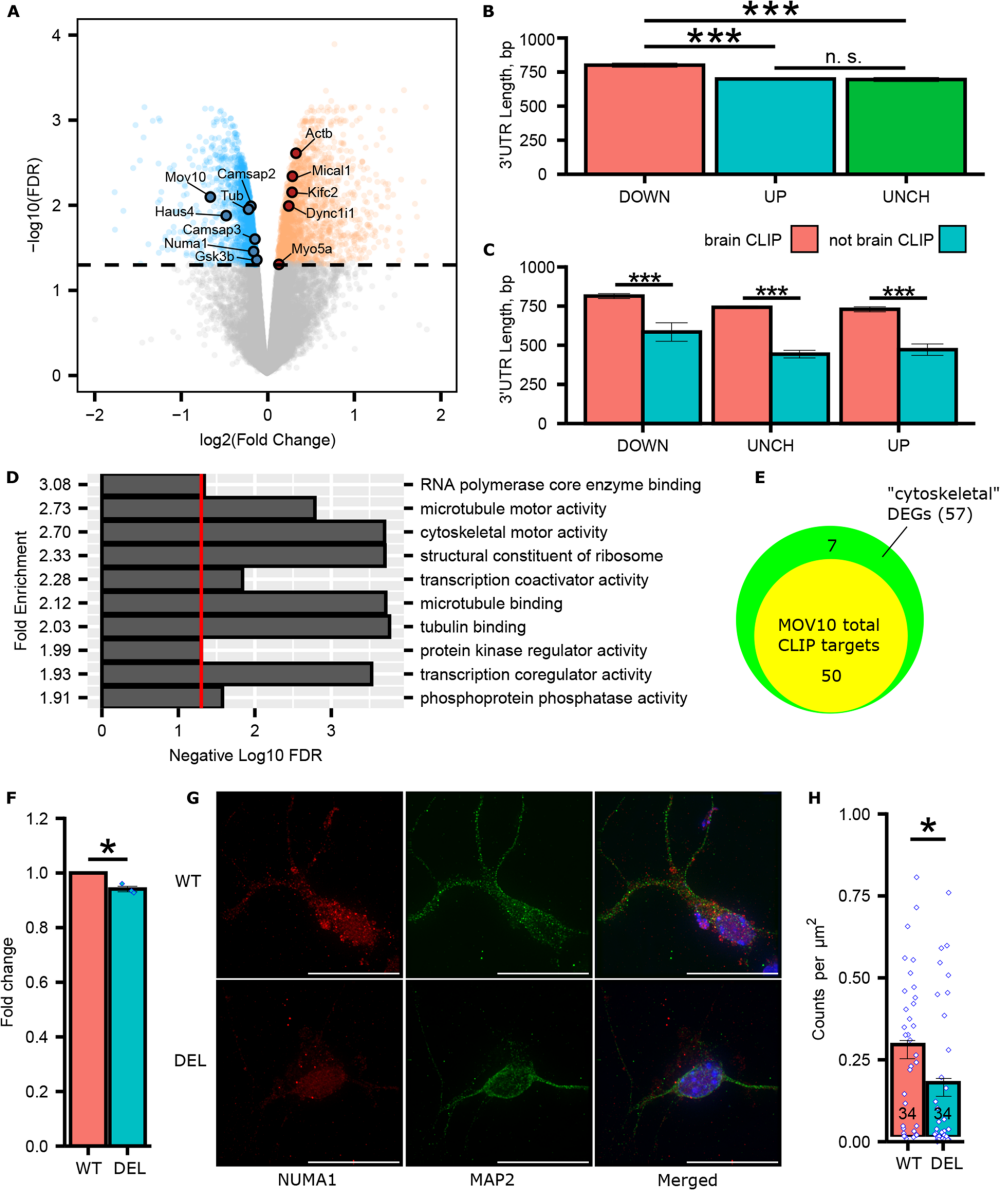
Differentially expressed mRNAs in the *Mov10* Deletion hippocampus and reduced NUMA1 expression. **A** Volcano plot of differentially expressed genes (DEGs) in P0 hippocampi of *Mov10* Deletion mice: blue-significantly decreased DEGs; orange-significantly increased DEGs relative to WT. *Mov10* and several cytoskeletal RNAs are indicated (see Discussion). 25 genes with log2(fold change) > 2 and log2(fold change) < −2 and/or -log10(FDR) > 4 are listed in Additional File 5: Table S2. **B** 3’UTR length in base pairs (bp) of the significantly decreased DEGs (DOWN), the significantly increased DEGs (UP) and the unchanged DEGs (UNCH). *P*-values were calculated using ANOVA and Tukey HSD test. **C** 3’UTR length (bp) of MOV10 brain CLIP and not CLIP DEGs. *P*-values were calculated using two-sided unpaired Student’s T-test. **D** Top ten most enriched categories of the DEGs using PANTHER GO-Slim Molecular Function. Red line represents the -log_10_(0.05) = 1.3. **E** Venn diagram of DEGs identified as cytoskeletal and total MOV10 CLIP targets (see description in text). **F** *Numa1* expression by RT-qPCR in P0 WT and *Mov10* Deletion (DEL) hippocampi. *P*-values were calculated using two-sided unpaired Welch's t-test. **G** Representative images of the DIV7 WT and *Mov10* Deletion (DEL) hippocampal neurons stained for NUMA1 and MAP2. Scale bar = 20 μm. **H** Quantification of NUMA1 particles (counts/μm^2^) in WT and *Mov10* Deletion (DEL) hippocampal neurons. Data are shown as mean ± SEM. *P*-values were calculated using Mann–Whitney U test. *p-value < 0.05, **p-value < 0.01, ***p-value < 0.001. “n” = number of neurons in the genotypes indicated from *N* = 2 litters cultured separately

We performed RNA-sequencing on the hippocampi of P0 *Mov10* Deletion and WT pups. We chose this timepoint because MOV10 is highly elevated in brain [8] and it is the day on which the neurons are isolated for primary culture, with the process of dendritogenesis initiating in vitro upon plating. We obtained 1,088,379,642 total reads across six samples and mapped 815,947,858 reads to the reference genome spanning 16,995 genes. After differential gene expression (DEG) analysis, we found that 1,985 and 2,069 genes were significantly down- and upregulated, respectively, in the *Mov10* Deletion hippocampi compared to WT (Fig. 5A and Additional File 4: Table S1). This result was in agreement with our earlier analyses of the MOV10-dependent transcriptomes in HEK293 cells [4] and murine Neuro2A cells [8], which both showed significantly up- and down-regulated mRNAs.

MOV10 binds primarily in the 3’UTR [4, 31], which contains RNA stability elements. When we compared the total transcript length (Additional File 6: Fig. S4A) and 3’UTR lengths of downregulated, upregulated, and unchanged genes, we found that the length of the 3’UTR was greater in the downregulated genes (Fig. 5B) and in the brain MOV10 CLIP targets within all three DEG groups (Fig. 5C), which is expected since the longer the 3’UTR, the more regulatory regions it contains [31]. Thus, it is likely that the 3’UTR is the target of MOV10 mediated regulation.

To determine if the transcriptomic changes we observed came from a specific neuronal subtype in the hippocampus, we analyzed the single-cell sequencing data from the hippocampi of human embryos at a gestation time comparable to mouse P0 [32, 33] and extracted specific markers of major neuronal groups in the hippocampus. After comparing our DEG list to the list of the specific markers, we did not find a cell-type specific difference in expression between different neuronal subtypes (Additional File 7: Table S3), probably because the number of unique markers for each neuronal subtype was much smaller than the number of DEGs (Additional File 6: Fig. S4B). Thus, we were unable to determine whether MOV10 had a neuron-specific effect on transcripts.

We also examined our RNA-seq data for alternative splicing because a previous study showed that MOV10 depletion in developing spermatogonia perturbed splicing [34]. We found that 91 transcripts had a significant isoform switch, but we did not identify any significantly changed events genome-wide (Additional File 6: Fig. S4C). Out of these 91 transcripts, we found that 33 transcripts have differential expression (0.8%), and 50 transcripts are direct brain CLIP targets of MOV10 (0.3%, Additional File 6: Fig. S4D). Thus, we conclude that the role of MOV10 in alternative splicing is not as significant in postnatal hippocampus as it is in developing spermatogonia.

In earlier work, we found that MOV10 preferentially bound cytoskeletal-related RNAs in total brain from P0 and P1 mice [8]. In our examination of P0 hippocampal DEGs, gene ontology (GO) analysis also identified genes associated with microtubule motor activity, cytoskeletal motor activity, microtubule binding, and tubulin binding (Fig. 5D). We also know that transcription factors are among MOV10’s directly bound targets [5], thus, many of the DEGs are likely downstream or indirect targets of MOV10. To focus on the directly bound mRNAs, we examined the 57 cytoskeletal DEGs for all available MOV10 CLIP targets [4, 8, 31, 34–37] and found that 50 are bound by MOV10 (Fig. 5E), providing candidates for further analysis.

### Microtubule binding protein NUMA1 expression in neurons is MOV10-dependent

We examined our DEG list for potential genes that might participate in regulating microtubules. Studies of *Drosophila* sensory da neurons revealed that centrosomal proteins, which usually participate in mitosis are co-opted for new uses in post-mitotic neurons, namely in dendrite outgrowth [12]. Centrosomin (mouse CDK5RAP2*)* tethers microtubule nucleation events and biases the direction of microtubule polymerization away from the dendrite tips. This is antagonized by wee Augmin (mouse HAUS), which promotes anterograde polymerization such that in the absence of centrosomin, unregulated wee Augmin leads to increased dendritic arborization [12]. While *Cdk5rap2* expression levels did not change in the absence of MOV10, another HAUS antagonizer, NUMA1 (*Numa1*) [11], is decreased in the absence of MOV10. *NUMA1* is also a MOV10 iCLIP target in HEK293 cells [4] and in spermatagonia [34] and NUMA1 has been observed in the somatodendritic compartment of the neuron, where its levels increase during early dendritic differentiation [10]. In studies of spindle formation, NUMA1 was shown to directly bind microtubules, promoting microtubule nucleation and elongation [38, 39]. We hypothesized that the compromised microtubule formation in the *Mov10* Deletion neurons is caused by reduced levels of NUMA1. To test this hypothesis, we first verified that there was less *Numa1* mRNA in *Mov10* Deletion hippocampi compared to WT using RT-qPCR (Fig. 5F). We then examined protein levels because MOV10 also regulates translation of its bound mRNAs [4]. Although examination of hippocampal extracts showed no significant difference in NUMA1 levels (Additional File 8: Fig. S5A), immunostaining of cultured hippocampal neurons showed significantly reduced NUMA1 expression in the neuronal cell body and dendrites in the absence of MOV10 (Fig. 5G, 5H, Additional File 8: Fig. S5B). Since neurons in Fig. 5G were imaged at high magnification and without tiling, only proximal branches can be observed, which are significantly reduced in *Mov10* Deletion (Fig. 3A). We conclude that MOV10 is playing a role in dendritic branching by regulating the translation of the microtubule organizer NUMA1, which is a reported HAUS antagonizer [11]. Since HAUS knockdown (KD) in neurons has been shown to lead to decreased levels of acetylated tubulins [40], we suspect that the increased HAUS activity in *Mov10* Deletion neurons leads to the increased levels of acetylated tubulin that we observed in Fig. 4D.

### MOV10 regulates Numa1 post-transcriptionally

**Fig. 6:**
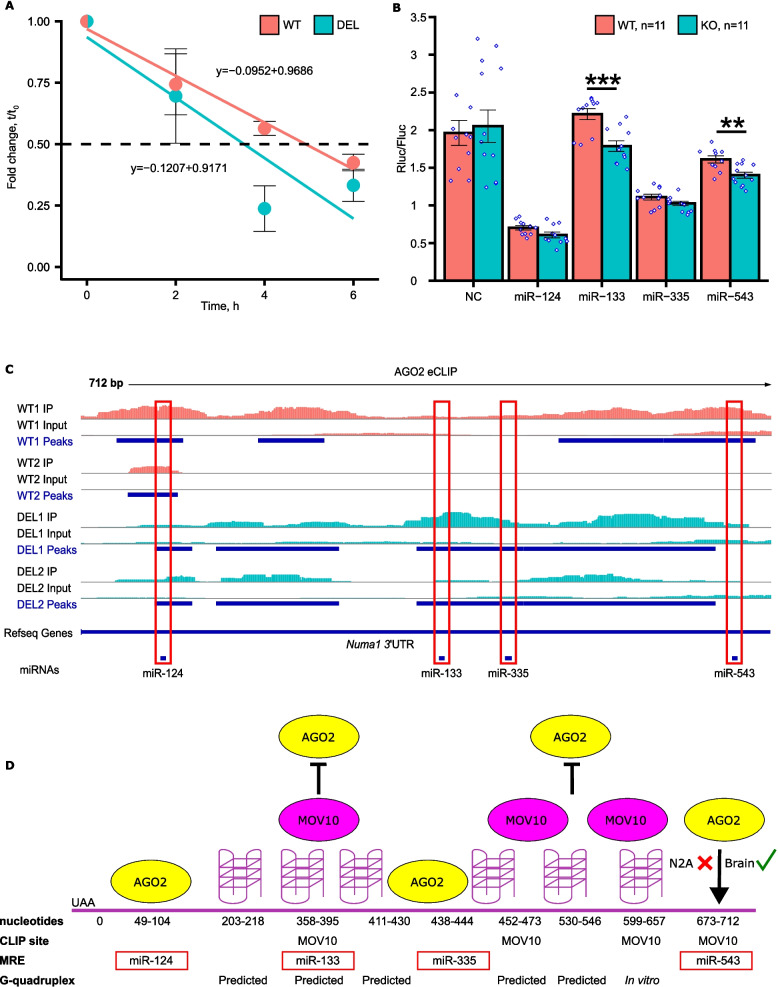
*Numa1* expression is post-transcriptionally regulated through its 3’UTR. **A** *Numa1* levels measured by RT-qPCR from WT and *Mov10* Deletion DIV4 hippocampal cultures treated with DRB. *Numa 1* half-life in WT = 4.93 h and in *Mov10* Deletion (DEL) = 3.54 h. **B** Luciferase expression (Renilla/Firefly luciferease) in Neuro2A WT and *Mov10* knockout (KO) cells transfected with psiCHECK-2-*Numa1*−3’UTR and either no miR (NC) or miR-124, −133, −335, and −543. Data are shown as mean ± SEM. *P*-values were calculated using Mann–Whitney U test. **p-value < 0.01, ***p-value < 0.001, *n* = 11, *N* = 3. **C** AGO2-eCLIP reads in *Numa1* from Immunoprecipitations (IP) from P0 WT brain (WT1 IP) (“Input” is total brain RNA) and two *Mov10* Deletion P0 brains DEL1 IP and DEL2 IP. WT2 IP is from a previously published AGO2 eCLIP [5]. Peaks (in blue) are the input-normalized clusters identified by CLIPper (v2.0.1). The track height range for all tracks is 0–30. Data are visualized in IGV (v2.18.0) and the location of the MREs are indicated below. **D** Model of *Numa1* 3’UTR (NM_001403544.1) with AGO2 eCLIP sites identified in (**C**) and MOV10 CLIP sites identified in [34], miR-124, −133, −335, and −543 miRNA recognition sites, and predicted G-quadruplexes [41] and an in vitro G-quadruplex indicated [42]. “X” indicates that MOV10 blocks miR-543 MRE in Neuro2A while in brain (checkmark) MOV10 appears to facilitate AGO2 association

mRNA levels are regulated at the level of transcription as well as post-transcriptionally in the cytoplasm through association with RBPs like AGO2. To determine if *Numa1* levels are decreased in the *Mov10* Deletion hippocampus because of reduced transcription, we treated DIV4 hippocampal neurons with the transcription inhibitor 5,6-dichloro-1-beta-D-ribofuranosyl-benzimidazole (DRB) and measured *Numa1* at 0, 2, 4 and 6 h by RT-qPCR (Fig. 6A). We observed that *Numa1* had a shorter half-life in *Mov10* Deletion neurons (3.54 h) compared to WT (4.93 h), suggesting that *Numa1* levels are regulated by a post-transcriptional degradation event that is blocked by the presence of MOV10. A similar observation was made for MOV10 target mRNAs with G-quadruplexes in their 3’UTRs [4], specifically, that MOV10 blocked AGO2 association.

The 3’UTR of murine *Numa1* is 716 nucleotides long with four predicted MREs in TargetScan: miR-124 is predicted to bind in the proximal region 49 nucleotides from the stop codon; miRs-133, −335 and −543 are predicted to bind in the distal half of the *Numa1* 3’UTR, which is also guanine-rich and predicted to form multiple G-quadruplexes [41]–one of which was experimentally identified in mouse embryonic stem cells (nucleotides 599–657, labeled in vitro) [42]. In addition, examination of our previously published AGO2 enhanced CLIP (eCLIP) data from P0 mouse brain identified *Numa1* as an AGO2 CLIP target, binding the miR-124 miRNA Recognition Element (MRE) in the 3’UTR (Table S1 in [5]). In further support of *Numa1* being regulated by AGO2, *Numa1* was recently shown to be regulated by miR-124 in axonal growth cones [43].

To further explore miRNA-mediated regulation of *Numa1*, we expressed its 3’UTR in a luciferase reporter in the presence or absence of MOV10 in WT Neuro2A cells and *Mov10* knockout Neuro2A cells [8]. We saw no difference in expression (Fig. 6B). This result was not surprising because endogenous *Numa1* expression is unchanged in the RNA-seq of WT and *Mov10* knockout Neuro2A cells (Table S9 in [8]). However, over-expression of a phospho-mimic of MOV10 (S970D) resulted in a significant reduction of endogenous *Numa1* (Table S1 in [35]), suggesting phospho-MOV10-dependent recruitment of AGO2. We next introduced the miRNAs predicted to regulate *Numa1* and examined their effect on reporter expression. Introduction of miR-124 suppressed expression, irrespective of whether MOV10 was present or not (Fig. 6B). This lack of MOV10 dependence is not surprising because there are no MOV10 CLIP sites in or around the miR-124 MRE nor are there predicted G-quadruplexes, which MOV10 binds to modulate AGO2 access [4, 5]. A similar result was obtained with miR-335, which suppressed the reporter in both WT and MOV10 knockout cells and its MRE was not in MOV10 CLIP sites [34]. In contrast, introduction of miR-133 led to a significant reduction in the absence of MOV10, suggesting that MOV10 blocks AGO2 access to the miR-133 MRE. This region is G-rich, with predicted G-quadruplexes, and was identified as being directly bound by MOV10 in a CLIP experiment [34]. Addition of miR-543 suppressed both WT and MOV10 knockout cells but there was more suppression in the absence of MOV10. Thus, MOV10 had a protective effect on MREs in or proximal to the G-rich regions the 3’UTR, as we have observed before [4, 5].

To confirm the protective role of MOV10 in regulating *Numa1* expression, AGO2 eCLIP was performed on WT and *Mov10* Deletion P0 brains [44, 45]. Most of the miRNA peaks were assigned to miR-124-3p, miR-9-5p, miR-125b-5p, and miR-466i-5p (Additional File 9: Fig. S6A). Interestingly, all these miRNAs are known to regulate different pathways in the brain [46–49]. As for AGO2 peaks, there are 22 genes with significantly enriched peaks in WT and 162 genes with significantly enriched peaks in *Mov10* Deletion (Additional File 9: Fig. S6B), suggesting that the primary role of MOV10 in the brain is to protect mRNAs. While AGO2 peaks on *Numa1* mRNA did not meet the enrichment cut-off, we observed differential binding of AGO2 to *Numa1* 3’UTR. Specifically, three major peaks, indicated by the blue lines in Fig. 6C, were observed on the *Numa1* 3’UTR in WT brain (WT1 IP), one of which was also observed in the earlier AGO2 eCLIP sample, WT2 [5, 50]. This AGO2 binding site contains the MRE for miR-124. In the absence of MOV10 (DEL), there is still AGO2 binding in this first region but to a lesser extent, supporting the experiment shown in Fig. 6B that miR-124 suppresses expression in a MOV10-independent manner. The second region of the 3’UTR is bound by AGO2 in the presence and absence of MOV10, although the AGO2 binding is more extensive, meaning a larger region in the 3’UTR, in the absence of MOV10 (DEL). There are no known MREs in the second region. The third AGO2 eCLIP peak is also much broader in the IPs from *Mov10* Deletion compared to the WT1 IP, suggesting increased AGO2 binding in the region containing MREs for miR-133 and −335 in the absence of MOV10, supporting the hypothesis that MOV10 protects this region from AGO2 association. These data also support the result in Fig. 6B where addition of miR-133 increased *Numa1* 3’UTR suppression in the absence of MOV10. In addition, the third region extends more distally in WT to include the MRE for miR-543, suggesting MOV10 facilitates AGO2 association in WT brain. This result is in contrast to Fig. 6B, which suggested MOV10 blocked AGO2 association with the MRE for miR-543. We suspect that the regulation of *Numa1* by MOV10 at miR-543 is cell-type specific, such that in Neuro2A, MOV10 protects *Numa1* and in neurons MOV10 facilitates AGO2 binding.

Figure 6D is a compilation of the AGO2 eCLIP data (Fig. 6C), the published MOV10 CLIP sites [34], predicted G-quadruplexes [41] and an in vitro verified G-quadruplex [42] to demonstrate how AGO2 associates with the 3’UTR of *Numa1* and how MOV10 association with G-quadruplexes blocks AGO2 association. As a result, we can see that the MREs for miR-124 and miR-335 do not have G-quadruplexes nor MOV10 CLIP sites, which is why their regulation is largely MOV10-independent. The MRE for miR-543 is bound by MOV10 but its involvement in regulation of *Numa1* is likely cell-type specific. Importantly, the MRE for miR-133 is predicted to have a G-quadruplex, which is recognized by MOV10 to regulate AGO2 association, allowing MOV10 to act as a protector of *Numa1*.

### Over-expression of NUMA1 or knockdown of HAUS6 rescues microtubule comet formation and decreases dendritic arborization in Mov10 Deletion neurons

Our model is that MOV10 controls *Numa1* expression by regulating AGO2 access to the 3’UTR. In the absence of MOV10, NUMA1 is decreased and unavailable to regulate HAUS, which leads to the dendritic phenotypes observed in *Mov10* Deletion neurons. To test our model for NUMA1-HAUS regulation of microtubule polymerization, we performed rescue experiments in *Mov10* Deletion neurons by introducing *Numa1* transgene or knocking down one of the HAUS proteins, which will disrupt the entire HAUS complex [40] and examining microtubule comet formation in dendritic growth cones. Specifically, we expressed EGFP-NUMA1 or *Haus6* shRNA in DIV0 *Mov10* Deletion neurons, after validation of their respective efficacies in Neuro2A cells (Additional File 8: Fig. S5C and D). As a control, we introduced an empty EGFP vector into the WT and *Mov10* Deletion neurons and all experimental groups were co-transfected with the mCherry-EB3 construct to allow measurements of comet formation and travel rate.

**Fig. 7:**
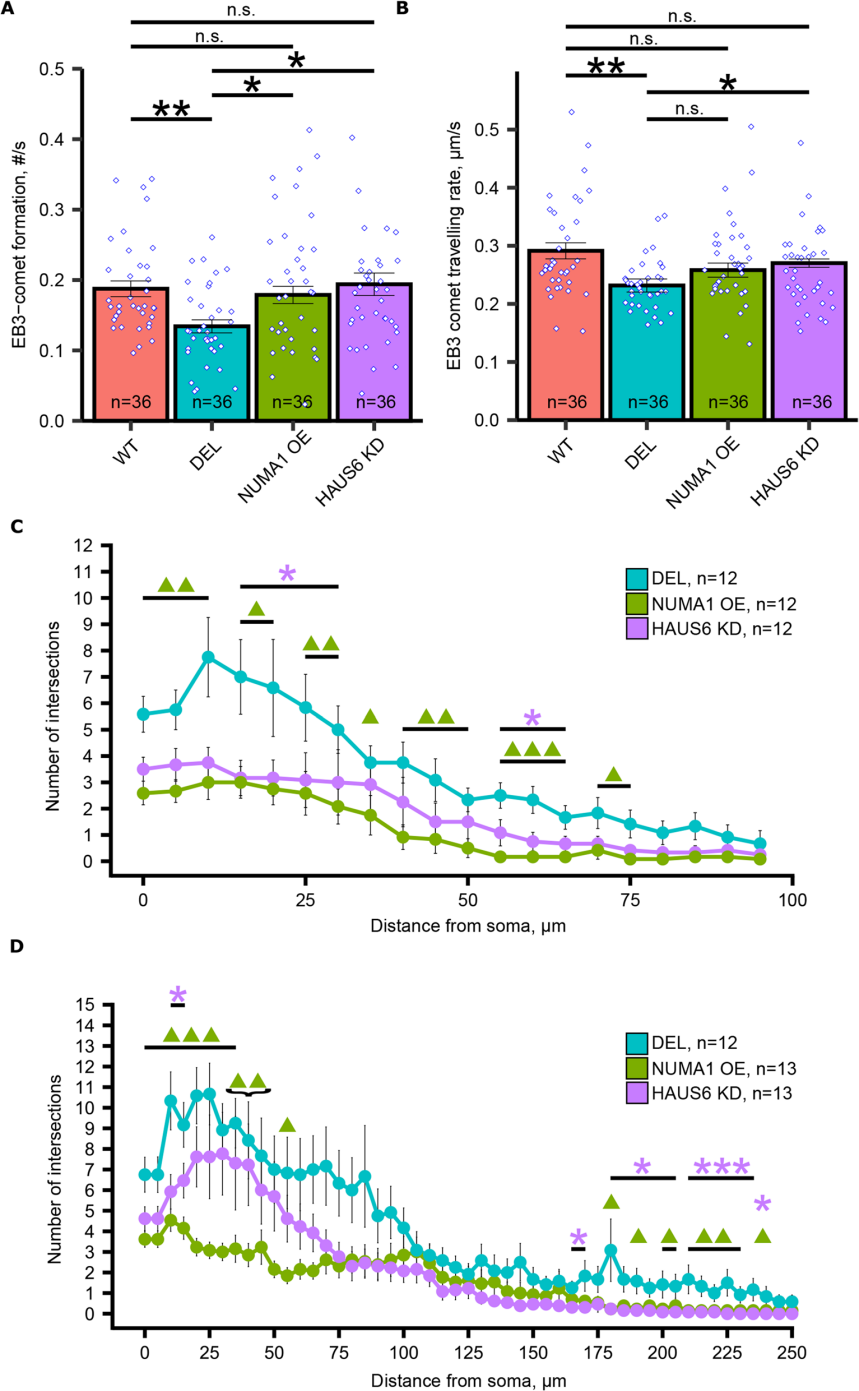
Over-expression of NUMA1 and reduction of HAUS6 in *Mov10* Deletion neurons rescues EB3 comet formation and reduces dendritic arborization. **A**, **B** DIV2 hippocampal neurons were transfected with EB3-mCherry plasmid and each of the following constructs: a control GFP plasmid pLenti-CMV-GFP-P2A-Puro into WT and *Mov10* Deletion (DEL) neurons; pEGFP-P2A-NUMA1 (NUMA1 OE) or SMART vector HAUS6 shRNA (HAUS6 KD) into *Mov10* Deletion neurons. EB3 comet formation (A) and EB3 traveling rate (**B**) were measured. **C** DIV0 *Mov10* Deletion hippocampal neurons were nucleofected with a control (pmaxGFP) (DEL), pEGFP-P2A-NUMA1 (NUMA1 OE), or SMART vector HAUS6 shRNA (HAUS6 KD) and Sholl analysis performed on DIV3. **D** DIV10 *Mov10* Deletion hippocampal neurons were transfected using Lipofectamine 2000 with a control (pmaxGFP) (DEL), pEGFP-P2A-NUMA1 (NUMA1 OE), or SMART vector HAUS6 shRNA (HAUS6 KD) and Sholl analysis performed on DIV10. Data are shown as mean ± SEM. *P*-values were calculated using Dunn’s test with Benjamini–Hochberg adjustment for multiple comparisons. *p-value < 0.05, **p-value < 0.01, ***p-value < 0.001, n is the number of neurons from *N* = 3 litters cultured separately. Purple asterisks indicate significant differences between *Mov10* Deletion and HAUS6 KD and green triangles indicate significant differences between *Mov10* Deletion and NUMA OE

Once again (as shown in Fig. 4), we found that both EB3-comet formation and comet traveling rate were significantly reduced in the *Mov10* Deletion neurons compared to WT (Fig. 7A and B). Importantly, over-expression of NUMA1 (NUMA1 OE) rescued microtubule comet formation in the *Mov10* Deletion neurons to WT levels and partially rescued the microtubule dynamics because the EB3-comets traveled faster than in the *Mov10* Deletion but is not significant, although the rate was indistinguishable from WT (Fig. 7D, n.s.). Thus, introduction of NUMA1 fully rescues comet formation and partially rescues the rate of travel. In contrast, knockdown of *Haus6* (HAUS6 KD) in the *Mov10* Deletion neurons led to the restoration of WT levels of both the EB3-comet formation rate and the EB3-comet traveling rate (Fig. 7A and B), likely because one of the proteins in the complex (HAUS4) is already down-regulated in *Mov10* Deletion neurons (Fig. 5A), thus, knockdown of HAUS6 further disrupts a reduced HAUS complex. We conclude that over expression of the HAUS complex participates in the *Mov10* Deletion phenotype in dendritic growth cones, supporting our hypothesis that normal dendritogenesis requires a balance of NUMA1 and HAUS expression (Fig. 7C).

To test this hypothesis in developing *Mov10* Deletion neurons, we used the same constructs as above, introducing them by nucleofection in freshly isolated DIV0 neurons and to transfect adherent DIV7 neurons to examine the effect on arborization using Sholl analysis. It became quickly apparent that the transfected neurons could not survive until complete maturity at DIV14 due to either high levels of exogenous gene expression or transfection cytotoxicity. Thus, we performed the Sholl analysis three days post-introduction, DIV3 and DIV10, respectively, when the neurons were still healthy. Both approaches led to a significant reduction in the distal dendritic arborization of *Mov*10 Deletion neurons in which NUMA1 was over-expressed and HAUS6 was knocked down. (Fig. 7B, C), supporting our hypothesis that MOV10 modulates NUMA1 and HAUS expression. Interestingly, overexpression of NUMA1 at both timepoints led to significantly reduced primary dendrites, whereas knockdown of HAUS6 at DIV7 led to a milder phenotype (Fig. 7C, D and Additional File 9: Fig. S6 C, D).

**Fig. 8:**
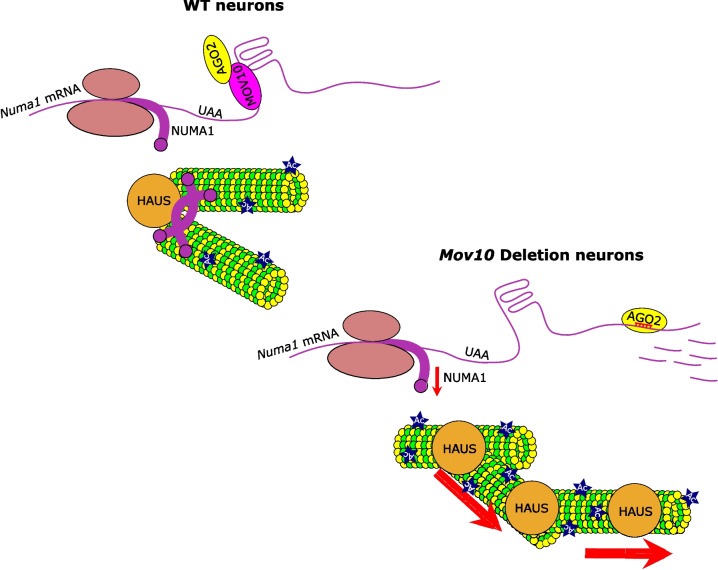
Model for MOV10 regulating access of AGO2 to the *Numa1* mRNA in neurons where NUMA1 and HAUS polymerize microtubules for normal dendritic branching. *Numa1* mRNA is translated into NUMA1. MOV10 binds the RNA G-quadruplex in the 3’UTR to prevent AGO2 from suppressing translation. NUMA1 and HAUS bind microtubules (green and yellow structures) to facilitate dendritogenesis. The asterisk indicates acetylation. In the absence of MOV10 (shown below), AGO2 accesses the MREs in the 3’UTR of the *Numa1* mRNA leading to translation suppression (downward red arrow) and subsequent degradation of the mRNA. In the absence of NUMA1, unregulated HAUS complex facilitates increased polymerization (thick red arrows) of microtubules

The rescue of the distal arborization phenotype suggests that the dysregulated interplay of NUMA1 and HAUS is at least partially responsible for the increased dendritic arborization in the absence of MOV10 (Fig. 8). However, decreased arborization of proximal dendrites by NUMA1 OE and HAUS6 KD is a further exacerbation of the *Mov10* Deletion phenotype shown in Fig. 3A. We suspect that MOV10 is responsible for regulating multiple mechanisms for dendrite development, and while NUMA1 and HAUS complex play an important role in the distal dendrites, their effect on proximal dendrites is outweighed by other MOV10-dependent pathways, addressed more in the Discussion.

In summary, we have identified a novel role for MOV10 in regulating translation of microtubule polymerizing protein NUMA1 for normal dendritogenesis and ultimately, for normal fear learning and memory.

## Discussion

MOV10 plays a crucial role in early embryonic development [8, 9]. Because of its elevated levels in postnatal brain [8] and its association with FMRP [4], we hypothesized that it also plays an important role in dendritogenesis and ultimately in learning and memory. To test this hypothesis, we created the *Mov10* Deletion mouse. We expected to find a phenotype because loss of MOV10 in tadpoles led to an abnormal distribution of neuronal precursor cells and mature neurons in the ventricular zone, suggesting abnormal proliferation, migration and/or increased maturation of neurons [9]. In addition, knockout mice of the MOV10-associated proteins FMRP and AGO2 both have behavioral phenotypes. Loss of FMRP leads to impaired learning and memory, similar to that observed in Fragile X syndrome [51]. In contrast, loss of functional AGO2, i.e., loss of miRNAs at the same times during development as the *Mov10 Deletion* mouse (both mice were created with the *Emx1*-Cre line) resulted in a smaller cortex, no detectable hippocampus and death by postnatal day 30 [30]—all much more severe than the *Mov10* Deletion phenotype. Thus, loss of brain miRNAs has a much more severe impact on brain development than the loss of MOV10. In fact, not only was the *Mov10* Deletion mouse viable into adulthood, it also had increased cortical thickness and a morphologically normal hippocampus. In contrast, conditional deletion of miRNAs in an adult mouse forebrain led to features more similar to the *Mov10* Deletion mouse, showing increased memory that included both fear and spatial memory as well as increased distal branching of hippocampal neurons in slices [23]. Thus, at the later time points, MOV10 loss and AGO2 loss are more similar. MOV10 likely modulates AGO2 association with mRNAs in arborizing hippocampal neurons, such that when each is deficient, the result is elongated distal dendrites and enhanced memory. It is possible that the *Mov10* Deletion mouse did not show enhanced memory in tests requiring normal vision because of MOV10’s possible role in retinal development, as observed in tadpoles treated with *Mov10* morpholinos [9]. Importantly, we show here that MOV10 plays an important role in fear memory extinction, which utilizes multiple brain regions including amygdala, cortex and hippocampus [52]. Our results suggest that MOV10-mediated translation regulation affects neuronal connectivity and at the molecular level, MOV10 is likely mediating many of its effects by modulating AGO2 access to mRNAs, thus, affecting translation of neuronal mRNAs.

Although we showed that the dendritic growth cone defect in the *Mov10* Deletion hippocampal neurons could be rescued by modulation of either NUMA1 or HAUS, we still do not know how these microtubule binding proteins interact. The rescue effect of HAUS6 downregulation in growth cones appears to be more profound than NUMA1 over-expression, possibly because one of the components of the HAUS complex, HAUS4, is downregulated endogenously in *Mov10* Deletion neurons. Further, previous reports showed that downregulation of HAUS1, HAUS6, and HAUS7 leads to reduced dendritic arborization [40, 53]. In addition, overexpression of NUMA1 was unable to completely rescue EB3 comet traveling rate and neither overexpression of NUMA1 or knockdown of HAUS6 were able to rescue the reduced primary branching. We hypothesize that MOV10 is regulating the expression of multiple cytoskeletal related proteins. MOV10 DEG candidates include the NUMA1 interacting protein KATNB1 [54] and microtubule binding and assembly proteins CAMSAP2 and CAMPSAP3 [55, 56], which are also reduced in the absence of MOV10 (Fig. 5A). MOV10 target mRNAs also encode proteins that destabilize microtubules like SPASTIN, which is among the DEGs reduced in the *Mov10* Deletion. Reduced SPASTIN could also explain the increase in stabilized microtubules we observed.

A recent study identified reduced NUMA1 expression in axonal growth cones as causing the developmental problems observed in Huntington’s disease (HD), leading to microtubule disorganization [43]. Introduction of miR-124 phenocopied the HD axonal defect caused by the huntingtin protein (HTT). Although it was not determined how HTT suppresses NUMA1 expression, our work begins to identify the RNA binding proteins that regulate NUMA1 expression in growth cones. MOV10 binds RNA G-quadruplexes to block access to AGO2 [4, 5]. The 3’UTR of *Numa1* is G-rich and is predicted to form a number of G-quadruplexes in the distal half of the transcript. In CLIP studies, MOV10 bound the MREs for miR-133 and miR-543 [34] and in our experiments, MOV10 blocks degradation of the reporter after their introduction. We suspect that regulation of these MREs could have important implications for dendritic development because miR-133 and −543 have been implicated in neurite outgrowth [57] and neuronal differentiation [58, 59], respectively.

In summary, we have identified a role for MOV10 in suppressing fear memory formation since the absence of the *Mov10* gene leads to enhanced fear memory. This may have implications for understanding Post Traumatic Stress Disorder (PTSD) and could give insight into Savant syndrome, a feature in some individuals with autism who have remarkable memory capacity [60]. In addition, individuals with autism have been reported to have increased cortical volume early in development [61] that is region specific [62]. We do not know why there is increased cortical thickness in the *Mov10* Deletion mouse, although it could be a result of increased dendritic arborization of the cortical neurons. Further studies are necessary to determine how cytoarchitecture affects complex behaviors.

## Conclusions

In this study, we established a role for the RNA helicase MOV10 in neuronal development and function through creation and characterization of a brain-specific knockout mouse, *Mov10* Deletion. Loss of MOV10 from excitatory *Emx1*-expressing neurons lead to enhanced fear memory, increased cortical thickness and increased dendritic arborization of hippocampal neurons. RNA-seq of P0 hippocampi revealed a preponderance of cytoskeletal-related mRNAs as MOV10-dependent, including the microtubule binding protein NUMA1. *Numa1* is protected from miRNA-dependent degradation by MOV10 and over-expression of NUMA1 and knockdown of *Haus6* in *Mov10* Deletion hippocampal neurons partially rescues the dendritic phenotype. Our work suggests that MOV10 regulates microtubule-mediated dendritic arborization with implications for normal forgetting.

## Methods

### Mouse husbandry

Mice were housed in standard IVC cages with water and food (Inotiv, Teklad, cat. #2918) available ad libitum. Mice were kept on a 12-h light/dark cycle from 7 AM to 7 PM and 7 PM to 7 AM, respectively. All experiments involving mice were reviewed and approved by the University of Illinois at Urbana-Champaign Institutional Animal Care and Use Committee based on the recommendations of the Guide for the Care and Use of Laboratory Animals of the National Institutes of Health. Credentials of the IACUC committee include USDA Registration: #33-R-0029; PHS Assurance: D16-00075 (A3118-01); AAALAC: #00766. Experiments were approved in IACUC protocols 19,112 and 22,113 (07/28/2022–2025). Mice were euthanized by carbon dioxide asphyxiation by replacing air in their home cage with CO_2_ at the rate of 70% volume/min until they ceased moving and stopped breathing. Death was confirmed by cervical dislocation. For neuron dissection, P0 pups were euthanized by decapitation.

### Creation of the Mov10 Deletion mouse

A detailed description of creation of this mouse is in the Supplemental Materials. Briefly, a *Mov10* targeting construct (*Mov10*^tm456285a(L1L2_GT2_LF2A_LacZ_BetactP_neo)^) was obtained from the European Mouse Mutant Cell Repository (EMMCR) [13] (https://www.i-dcc.org/imits/targ_rep/alleles/43670/targeting-vector-genbank-file) that targeted two exons (6 and 7) that when removed from the mouse *Mov10* gene, leads to loss of frame. We verified the targeting construct by sequencing and introduced it into C57BL/6 J ES cells by electroporation to obtain 232 neomycin-resistant clones. DNA was extracted and screened for successful insertion into the *Mov10* locus using PCR (primers described in Supplementary Material) which identified 10 clones that were correctly targeted. The presence of all LoxP sites was verified by sequencing. Three of the correctly targeted clones were introduced into albino blastocysts for chimeric animal production. The chimeras were crossed to albino females and the resulting black pups were screened by PCR for the presence of the targeting construct (by identifying the neomycin cassette) and were then bred to homozygosity. These mice were then crossed with the FLP transgenic mouse (009086) from Jackson Laboratories to remove the neomycin cassette (flanked by FRT sites), which can affect the phenotype [63]. The FLP transgene is on chromosome 6: since the targeted *Mov10* locus is on chromosome 3, this experiment worked. Successful removal of the neomycin cassette was monitored by PCR (Additional File 10). To delete MOV10 expression from brain, we obtained the *Emx1-Cre* mouse (005628) from the Jackson Laboratory, which begins low expression in neurons at E10.5 but steadily increases [16] to obtain brain-specific-*Mov10* knockouts (*Mov10* cKO). After observing only a 50% reduction of MOV10 in hippocampus, we bred the *Mov10* cKO with the previously described *Mov10* heterozygous mutant (*Mov10* HET) mouse to obtain the novel mouse model we called *Mov10* Deletion. The Western blotting analysis of P0 brain lysates from *Mov10* Deletion mouse showed a 90% reduction of the MOV10 protein level.

### Western blotting

Hippocampi from at least two P2 WT and *Mov10* Deletion per pups per sample were dissected, lysed in lysis buffer (50 mM Tris–Cl 7.5, 300 mM NaCl, 30 mM ethylenediaminetetraacetic acid (EDTA), 0.5% Triton), quantified by Bradford assay, and resuspended in 1 × sample buffer for resolution by SDS-PAGE (7.5% gels) and analyzed by immunoblotting. Briefly, membranes were blocked with 5% non-fat dry milk in phosphate-buffered saline (PBS) containing 1% TWEEN-20 for 1 h at room temperature. Primary antibody was applied overnight at 4 °C followed by a brief wash in 1% non-fat milk PBS containing 1% TWEEN-20 wash buffer. Horseradish peroxidase (HRP)-conjugated secondary antibody was applied at 1:5000 dilution for 1 h at room temperature and washed 4 × 15 min using wash buffer. The HRP signal was detected using an enhanced chemiluminescent (ECL) substrate on BioRad ChemiDoc. The primary antibodies used were anti-MOV10 (RRID:AB_1040002, A301-571A, Bethyl Laboratories, Montgomery, TX, USA) at 1:1000, anti-eIF5 (RRID:AB_631427, sc-282, Santa Cruz Biotechnology) at 1:4000, anti-NUMA1 (NB500-174SS, Novus Biologicals) at 1:500, anti-acetylated tubulin (T7421-25UL, Sigma-Aldrich) at 1:1000, anti-GAPDH (RRID:AB_307274, ab9484, Abcam) at 1:5000, and HRP-conjugated goat anti-rabbit (RRID:AB_2337937, 111–035-008, Jackson Immunoresearch) and goat anti-mouse (RRID:AB_2338512, 115–035–174, Jackson Immunoresearch). The density of the bands was quantified using ImageJ. The level of significance and tests performed are described in the figure legends for each experiment.

### Whole mouse brain fixation, sectioning, and staining

Brains of 12-week-old mice were fixed in 4% paraformaldehyde in PBS overnight at 4 °C and then washed in a series of ethanol solutions for 30 min (25%, 50%, 70%, 83%, 95%, and 100%) and left in methyl salicylate overnight before embedding in paraffin. 7 μm coronal sections (Allen Brain Reference Atlas, Adult Mouse, Image 74) were prepared using a Leica RM2255 rotary microtome and dried overnight at room temperature. The sections were de-paraffinized using xylene and rehydrated through a series of ethanol washes (100%, 95%, and 70% followed by water) before staining with hematoxylin and eosin. The slides were imaged on NanoZoomer Slide Scanner and 10 × magnified fields of view were exported as TIFF images using NDP.view 2. TIFF images were anonymized prior to the analysis of the cortical and hippocampal layers. Quantification of thickness was done using the straight-line tool in ImageJ. Before quantification of cell density, the image was auto-thresholded using the Phansalkar method, despeckled, eroded, and dilated, and then the cell density was quantified using the analyze particle function (size 20 – infinity).

### Behavior tests

Mice aged 8–12 weeks old were tested at the same hour of the day in the following sequence: elevated plus maze, open field, novel object recognition, cued and context fear conditioning. Y-maze, and T-maze were performed on a separate cohort. The experimenter was blinded to the genotypes. Both sexes were tested. All trials for each mouse were videotaped with a Logitech HD Pro webcam and analyzed in TopScan, Cleversys Inc. software. Mixed-effect ANOVA was used as a statistical test to account for effects of sex and batch on the differences between the genotypes.

### Elevated plus maze

The apparatus consists of four arms (66 × 6.4 cm), an open area in the center (6.4 cm), two opposing open arms, and two opposing closed arms (20-cm-high wall) with sliding doors at the end. The maze is elevated at a height of 60 cm from the floor and was constructed by the Machine shop of the School of Molecular and Cellular Biology, UIUC. Mice were placed in the center of the maze and allowed to explore the maze for 10 min. Time spent in each zone was measured and used for the analysis.

### Open field test

The test was performed on the first day of the novel object recognition test. Mice were exposed for 10 min to a rectangular arena (46 × 25 × 20 cm), and the distance covered was measured and used for the analysis.

### Novel object recognition

The test was performed as described [64]. Briefly, mice were habituated to the empty arena on the first day for 10 min. After 24 h, two similar objects were presented, and the interaction with each object was tracked using a webcam. The pair of objects used in the test was randomized between animals. On day 3, a novel object replaced one of the objects, and the mice were recorded. The placement of the novel object was randomized between animals. The videos were analyzed to estimate the time the animal spent interacting with the objects that was used for the analysis.

### Cued and context fear conditioning

A modified procedure of the test was performed as described [23]. Mice were trained by exposing them for 5 min to a chamber (34 × 28 × 30 cm) where they received three consecutive pairs of tone (20 s) and shock (0.5 mA, 2 s) with an empty trace interval of 1 s and a 3-min break between each tone-shock pairing. One day later, behavior was recorded in a novel context using a webcam with the same tone but without shock (cued fear conditioning). Two days after training, the mice are placed back in the original training chamber without tone or shock and recorded for 8 min (context fear conditioning). Freezing was defined as lack of movement for at least 1 s, except for respiration. Percent of time spent freezing was used for the analysis.

### Y maze (YM) spontaneous alternation test

A mouse was placed in the Y-shaped maze built from grey plastic material with three arms (ABC) of equal size at a 120-degree angle from each other (arms: 30 cm long, 14.5 cm high, 7 cm wide) and allowed to freely explore the three arms for 5 min, and each movement from one arm into another arm was recorded. The percentage of alternation between the arms entered was used for the analysis.

### T-maze (TM) rewarded alternation

Mice were diet-restricted for two weeks before the test to maintain 90 ± 5% of initial weight. During this time, the animals were habituated to chocolate morsels that were used as food rewards. On day 1 (habituation), a mouse was placed in the maze with food rewards present in both goal arms for 10 min to let the animal habituate to the maze. On day 2 (training), one goal arm was closed by a door and the reward was placed in the other goal arm. A mouse was placed at the base arm and allowed to explore the open goal arm and consume the reward. The reward was refilled in the opposite goal arm, and the block was removed. Then the mouse was placed in the base arm so it could choose between the two goal arms. The mouse was allowed to consume the reward if it chose the correct arm or removed after a time equivalent to that normally used to consume the reward if it chooses the incorrect arm. This trial was repeated 10 times in a row for each animal. The percentage of correct trials was used for the analysis.

### RNA sequencing and analysis

Three samples for both WT and *Mov10* Deletion were prepared by isolating RNA using an RNeasy Mini Kit (Qiagen) from hippocampi of at least two P0 pups per sample. The quality of the isolated RNAs was checked using gel electrophoresis, and the RNAs were sent to the UIUC Roy J. Carver Biotechnology Center. RNA-seq libraries were prepared using TruSeq Stranded mRNAseq Sample Prep kit (Illumina) and 150 bp reads were generated using NovaSeq 6000 SP flowcell (Illumina). After adapter trimming and quality checking using multiQC (v1.9), abundance of each transcript was quantified using the Selective Alignment method of Salmon (v1.4.0) with a decoy-aware transcriptome using the entire GRCm39 genome as the decoy. Estimated feature expression levels were normalized using TMM (trimmed mean of M values) normalization in the edgeR (v3.32.1) package with the detection threshold at 0.25 cpm (counts per million) in at least 3 samples. RUVSeq (v1.24.0) package was used for the surrogate variables analysis to control the effects of outlying samples. Differential gene expression (DE) analysis was performed using the limma-trend method in limma (v3.46.0). Two surrogate variables calculated from RUVSeq were included in the statistical model as covariates to control unwanted noise. A one-way ANOVA test was calculated along with all three pairwise comparisons between the two groups. Multiple testing correction was done using the False Discovery Rate method. Alternative splicing was analyzed using the IsoformSwitchAnalyzeR (v 1.12.0). Analysis of the single-cell RNA-seq data from [33] was done using Seurat (v4.0.3).

### Hippocampal neuron culture

Hippocampal neurons of P0 pups were dissected and dissociated as described [65]. Coverslips were coated for 2 h at room temperature with 10 μg/mL of poly-L-lysine (P4707, Sigma) and 50,000 cells/well were plated for immunofluorescence (IF) in minimum essential medium (MEM) supplemented with 10% fetal bovine serum (FBS). After 24 h, the medium was switched to Neurobasal (NB) medium (21,103,049, Gibco) supplemented with B-27 (17,504–044, Gibco). Half of the media was removed and replaced with fresh NB medium every 3 days.

For live-imaging, 2*10^6^ hippocampal neurons were transfected using the Amaxa Nucleofector Device 2b with 3 μg of pCMV-EB3mCherry_cmv-LifeActGFP and placed on Poly-D-Lysine Coated glass-bottom 35 mm dishes (P35GC-1.5–14-C, MatTek). For the rescue of microtubule dynamics, the procedure was identical, but the neurons were transfected with 1.5 μg of pCMV-EB3mCherry and 1.5 μg of either pLenti-CMV-GFP-P2A-Puro, or pEGFP-P2A-NUMA1, or SMART vector HAUS6 shRNA (Dharmacon, V3SVMM01_16758209).

For the 5,6-dichloro-1-beta-D-ribofuranosyl-benzimidazole (DRB) experiment, 2*10^6^ hippocampal neurons from P0 pups were cultured in a 6-well plate for 4 days. On DIV4, the culture medium was adjusted to 1 mL and DRB (D1916-10MG, Sigma Millipore) was added to final concentration of 100 μM (3.3 μL of 30 mM DRB in DMSO). After 0, 2, 4, and 6 h following DRB addition, neurons were washed with 1 mL of HBSS, and total RNA was isolated using 1 mL (Invitrogen, cat. 15,596,026) following manufacturer’s protocol followed by RT-qPCR.

For the rescue of dendritic arborization, 2*10^6^ hippocampal neurons were transfected using the Amaxa Nucleofector Device 2b with 3 μg of pmaxGFP (Amaxa), pEGFP-P2A-NUMA1, or SMART vector HAUS6 shRNA (Dharmacon, V3SVMM01_16758209) and placed on Poly-D-Lysine Coated glass-bottom 35 mm dishes (P35GC-1.5–14-C, MatTek) and fixed 3 days after on DIV3 for imaging. Separately, 2.5*10^5^ hippocampal neurons were cultured on nitric acid etched coverslips coated with PDL. Neurons were transfected on DIV 7 with 0.8 µg pmaxGFP (Amaxa), pEGFP-P2A-NUMA1, or SMART vector HAUS6 shRNA (Dharmacon, V3SVMM01_16758209) using 2 µL of Lipofectamine 2000 (11,668,027, Invitrogen) per coverslip. On DIV 10, the neurons were fixed, immunostained with anti-MAP2 antibody, and imaged as described below.

### RT-qPCR

Total RNA was isolated from samples using TRIzol (Invitrogen, cat. 15,596,026) following manufacturer’s protocol. Total RNA was treated with TURBO DNase (Invitrogen, ref AM2238) for 30 min at 37 °C and heat-inactivated for 10 min at 75 °C. DNase-treated total RNA was converted to cDNA using M-MuLV Reverse Transcriptase (NEB, cat. M0253S) and Random Primer Mix (Promega, REF C1181) following manufacturer’s protocol with addition of 5 mM DDT. 5 ng of cDNA was used for RT-qPCR reaction using MicroAmp Fast 96-Well Reaction Plate (Applied Biosystems, ref 4,346,907), Cycler iQ Optical Tape (Bio-Rad, cat. 2,239,444), and PowerUp SYBR Green Master Mix (ThermoFisher, ref A25742) following manufacturer’s protocol on Applied Biosystems QuantStudio 3 system RT-qPCR machine. The following primer sets were used for RT-qPCR: Haus6, set 1 forward 5’-CAGTTCCACACTCCTTGAGAAGGATCC-3’, reverse 5’-CTACTCTGGCAACCTCATCTACCAGAC-3’, Haus6, set 2, forward 5’-TTCAAAAGGTTCGGTCCTTGTGGGC-3’, reverse 5’-CCTATCTGCAACCGACATATCTGCTCC-3’, Numa1 forward 5’-CCTGGCACTCCTGAGTCCAA-3’, reverse 5’-CCGGTCCGCCTGTTTGAGAA-3’, Gapdh forward 5’-CCGGGGCCCACTTGAAGG-3’, reverse 5’-TGGCATGGACTGTGGTCATGAGC- ‘3.

### Immunofluorescence and microscopy of cultured neurons

Neurons grown on coverslips were fixed in 4% paraformaldehyde for 10 min at room temperature on DIV 7 or DIV14. Samples were blocked in 10% normal donkey serum (017–000–121, Jackson ImmunoResearch) for 30 min at room temperature. Fixed neuron cultures were immunostained overnight at 4 °C with the following primary antibodies: anti-MOV10 (RRID:AB_1040002, A301-571A, Bethyl Laboratories) at 1:1000, anti-MAP2 (RRID:AB_91939, AB5622, Millipore) at 1:1000, anti-MAP2 (RRID:AB_2533001, 13–1500, Invitrogen) at 1:1000, anti-acetylated tubulin (T7-451, Sigma Aldrich) at 1:1000, and anti-alpha tubulin (RRID:AB_2288001, ab4074, Abcam). Alexa Fluor 594 goat anti-rabbit (RRID:AB_2307325, 111–585-144, Jackson ImmunoResearch) and Alexa Fluor 488 donkey anti-mouse (RRID:AB_2340846, 715–545-150, Jackson ImmunoResearch) secondary antibodies were added for 2 h at room temperature. Coverslips were inverted unto glass slides containing ProLong Gold Antifade Mountant with DAPI (P36930, Invitrogen) or in-house mounting media (0.3 μg/ml DAPI, 10% w/v Mowiol 4–88, 1% w/v DABCO, 25% glycerol, 0.1 M Tris, pH 8.5). Fluorescence images of DIV7 and DIV14 neurons were obtained with a Zeiss LSM 700 inverted confocal microscope using a 63 × EC Plan-Neufluar 1.40 oil objective. Images were captured with a cooled charge-coupled device (CCD) camera running Zen Black Software. A total of 9 tiles of 5 0.6-μM-thick sections were acquired as z-stacks. Fluorescence images of DIV7 neurons were obtained with DeltaVision OMX deconvolution microscope (GE Healthcare Life Sciences) with 100X 1.42 NA objective and EMCCD (Evolve) camera. A total of 31 0.2-μM-thick sections were acquired as z-stacks. Neurons cultures for rescue experiments were imaged on LSM900 using PlanApo 20X/0.8 air objective.

### Live microscopy

Neurons on 35 mm glass-bottom dishes were imaged at DIV2-4 using DeltaVision OMX deconvolution microscope (GE Healthcare Life Sciences) with 100X 1.42 NA objective and EMCCD (Evolve) camera. A total of 7 0.4-μM-thick sections were acquired as z-stacks for 2–3 min with 2–4 s frame rate. Images were processed using OMX Align and Deconvolution functions prior to analysis.

### Sholl analysis

All images were anonymized prior to the analysis. Confocal z-stacks were converted to planar images using sum slices projection and dendrites were traced using the SNT plugin in ImageJ. Sholl analysis was performed using the same SNT (v4.0.8) plugin in ImageJ. The radius step size was set at 5 μm.

### Microtubule and actin polymerization rate analysis

All images were anonymized prior to the analysis. EB3-comets were traced and analyzed using the MTrackJ plugin in ImageJ. Actin retrograde movement was analyzed using MTrackJ plugin in ImageJ.

### Luciferase assay

3’UTR of *Numa1* was subcloned from genomic DNA of C57Bl/6 J WT mouse using the following primers (forward 5’-ATCTCGAGACAGTCAGCACCAGTGCCTA-3’, reverse 5’-ATCCGGCCGCTCAAGGGAGAAAAATAGACTTTATTTAC-3’) and cloned into the psiCHECK-2 vector using NotI and XhoI. The resulting plasmid psiCHECK-2-*Numa1*−3’UTR was sequenced to verify correct insertion. For the Luciferase assay, WT and *Mov10* KO Neuro2a cells were seeded on Greiner Bio-One CELLSTAR 96-well plate (07–000–138, Fisher Scientific) 5*10^5^ cells/well. On the next day, cells were transfected with 0.4 μg/well of psiCHECK-2-*Numa1*−3’UTR, 0.5 μL/well of 4 μM stock of Dharmacon miRIDIAN microRNA (Mouse mmu-miR-124-3p-Mimic, cat C-310389–05-0002, Mouse mmu-miR-335-5p-Mimic, cat C-310609–05-0002, Mouse mmu-miR-543-3p-Mimic, cat C-310652–05-0002, Mouse mmu-miR-133a-3p-Mimic, cat C-310407–07-0002), and 0.5 μL/well Lipofectamine 2000 (Invitrogen, REF 11668–019) following manufacturer’s protocol. On the next day, luciferase activity was measured on Biotek Synergy 2 SL Microplate Reader using Luc-Pair Duo-Luciferase HT Assay Kit (GeneCopoeia, cat LF013) following the manufacturer’s protocol.

### eCLIP

The standard eCLIP protocol [66] was modified to enable chimeric ligation of miRNA and mRNA according to bioRxiv preprint [67]. Studies were performed by Eclipse Bioinnovations Inc. (SanDiego, www.eclipsebio.com) on submitted flash-frozen P0 brains. Mouse brain tissues were ground into a fine powder under liquid nitrogen, UV crosslinked twice at 400 mJoules/cm2 with 254 nm radiation, and stored until use at −80 °C. Cryoground tissue was then lysed with 750 μL of eCLIP lysis mix and sonicated (QSonica Q800R2) for 4 min, 30 s on / 30 s off with an energy setting of 75% amplitude, followed by digestion with RNase-I (Ambion). A primary mouse monoclonal AGO2/EIF2C2 antibody (sc-53521, Santa Cruz Biotechnology) was incubated for 1 h with magnetic beads pre-coupled to the secondary antibody (M-280 Sheep Anti-Mouse IgG Dynabeads, Thermo Fisher 11202D) and added to the homogenized lysate for overnight immunoprecipitated at 4 °C. Following overnight IP, 2% of the sample was taken as the paired size-matched input with the remainder magnetically separated and washed with eCLIP high stringency wash buffers. Chimeric ligation was then performed on-bead at room temperature for 1 h with T4 RNA ligase (NEB). IP samples were then dephosphorylated with alkaline phosphatase (FastAP, ThermoFisher) and T4 PNK (NEB) and an RNA adapter was ligated to the 3’ ends. IP and input samples were cut from the membrane at the AGO2 protein band size to 75 kDa above. Western blot was visualized using anti-AGO2 primary antibody (50,683-RP02, SinoBiological) at a 1:5000 dilution, with TrueBlot anti-rabbit secondary antibody (18–8816-31, Rockland) at 1:5000 dilution. RNA adapter ligation, IP-western, reverse transcription, DNA adapter ligation, and PCR amplification were performed as previously described.

After sequencing, samples were processed with Eclipsebio's proprietary analysis pipeline (v1). UMIs were pruned from read sequences using umi_tools (v1.1.1). Next, 3' adapters were trimmed from reads using cutadapt (v3.2). Reads were then mapped to a custom database of repetitive elements and rRNA sequences. All non-repeat mapped reads were mapped to the mm10 genome using STAR (v2.7.7a). PCR duplicates were removed using umi_tools (v1.1.1). AGO2 eCLIP peaks were identified within eCLIP samples using the peak caller CLIPper (v2.0.1). For each peak, IP versus input fold enrichments and *p*-values were calculated.

miRNAs from miRBase (v22.1) were "reverse mapped" to any reads that did not map to repetitive elements or the genome using bowtie (v1.2.3). The miRNA portion of each read was then trimmed, and the remainder of the read was mapped to the genome using STAR (v2.7.7a). PCR duplicates were resolved using umi_tools (v1.1.1), and miRNA target clusters were identified using CLIPper (v2.0.1). Each cluster was annotated with the names of miRNAs responsible for that target. Peaks were annotated using transcript information from GENCODE vM25 with the following priority hierarchy to define the final annotation of overlapping features: protein coding transcript (CDS, UTRs, intron), followed by non-coding transcripts (exon, intron).

### Statistical analysis

Data for each experiment was obtained independently through random sampling. Prior to statistical analysis, normality was assessed using the Shapiro–Wilk test, and homoscedasticity was evaluated via the F-test for comparisons involving two groups and Bartlett’s test for comparisons involving more than two groups. In cases where the assumption of normality was violated, the Mann–Whitney U test was employed for involving two groups, and Dunn’s test, with Benjamini–Hochberg correction for multiple comparisons, was applied for comparisons involving more than two groups. For data that met the normality assumption but exhibited unequal variances, Welch’s t-test was utilized for two-group comparisons. When both normality and homogeneity of variance were satisfied, Student’s t-test was used for two-group comparisons, and ANOVA was applied for comparisons across multiple groups, followed by Tukey’s HSD post-hoc test. All statistical analyses were conducted in R (v4.4.1). The lmerTest package (v3.1–3) was used for mixed-effects ANOVA, and the FSA package (v0.9.5) was employed for Dunn’s test.

## Supplementary Information

Additional file 1. Fig. S1. Cell densities in cortical layers and hippocampus and behavior tests.

Additional file 2. Fig. S2. Percentage freezing by minute during fear conditioning tests**.**

Additional file 3. Fig. S3. Phase-contrast images and distance travelled by EB3-comets, filopodia retraction rate, and acetylated tubulin in hippocampal extracts.

Additional file 4. Table S1. Differentially expressed genes in *Mov10* Deletion hippocampi.

Additional file 5. Table S2. Differentially expressed genes with log2(fold change)>2 and log2(fold change)<-2 and or -log10(FDR)>4.

Additional file 6. Fig. S4. Characteristics of the transcripts identified in RNA-seq**.**

Additional file 7. Table S3. Percentage of genes shared between neuron-specific markers and differentially expressed (DE) genes.

Additional file 8. Fig. S5. NUMA1 expression and efficiency of NUMA1 over-expression and *Haus6* knockdown. 

Additional file 9. Fig. S6. miRNA and AGO2 peaks identified in eCLIP and representative neurons.

Additional file 10. Creation of the *Mov10* Deletion mouse.

Additional file 11. Original uncropped Blots for Fig. 1B, Fig. S3D, and Fig. S5A.

## Data Availability

All data generated or analysed during this study are included in this published article, its supplementary information files and publicly available repositories. The RNA-seq and eCLIP data are available through GEO accession number GSE232771 and GSE278282, respectively. The rest of the data is available through Illinois Data Bank https://doi.org/10.13012/B2IDB-9303385_V1[68].

## Abbreviations

AGO2: Argonaute 2, an endonuclease directed to target mRNAs through a bound miRNA
APC: Apical Precursor Cell
DEG: Differentially expressed gene
DIV 14: Day in vitro 14
DRB: Inhibitor 5,6-dichloro-1-beta-D-ribofuranosyl-benzimidazole
EGFP: Enhanced green fluorescent protein
HD: Huntington’s disease
KD: Knockdown
KO: Knockout
miRNA: MicroRNA
mRNA: Messenger RNA
miR-124: MicroRNA-124
MOV10: Moloney Murine Leukemia Virus Insertion 10
MRE: MicroRNA recognition element
OE: Over-expression
P0: Post-natal day 0
PCR: Polymerase Chain Reaction
PTSD: Post Traumatic Stress Disorder
RBP: RNA-binding protein
RT-qPCR: Real time quantitative polymerase chain reaction
RNA: Ribonucleic Acid
Seq: Sequencing
SEM: Standard Experimental Mean
UTR: Untranslated region
WT: Wild Type (the normal control mouse without manipulation or engineering, C57Bl/6)
