# Supplementary figures and images for: RNA helicase MOV10 suppresses fear memory and dendritic arborization and regulates microtubule dynamics in hippocampal neurons
Source: BMC Biol. 2025 Feb 6;23:36. doi: 10.1186/s12915-025-02138-6 (PMC11803958; doi:10.1186/s12915-025-02138-6)

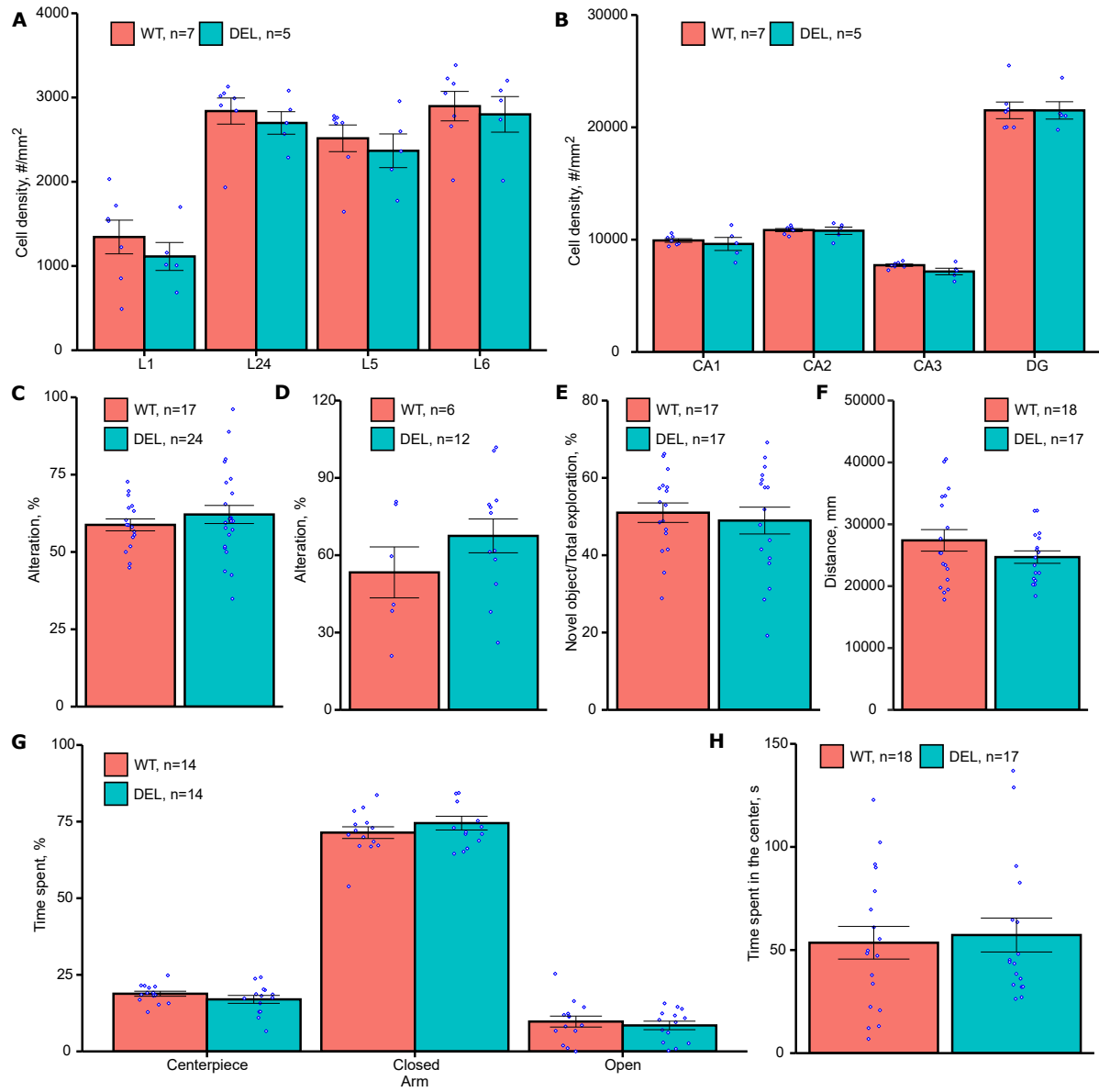

Supplement: Supplementary file 1 — Additional file 1. Fig. S1. Cell densities in cortical layers and hippocampus and behavior tests. [file 12915_2025_2138_MOESM1_ESM.pdf]

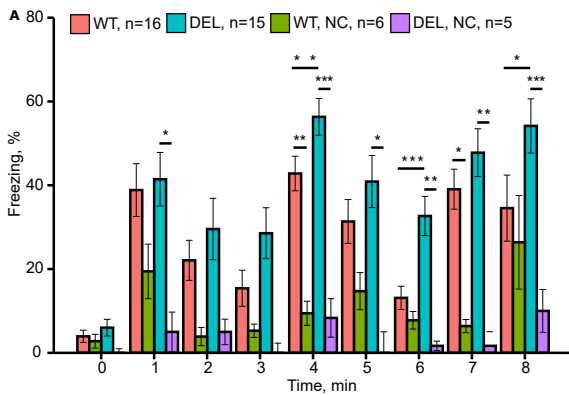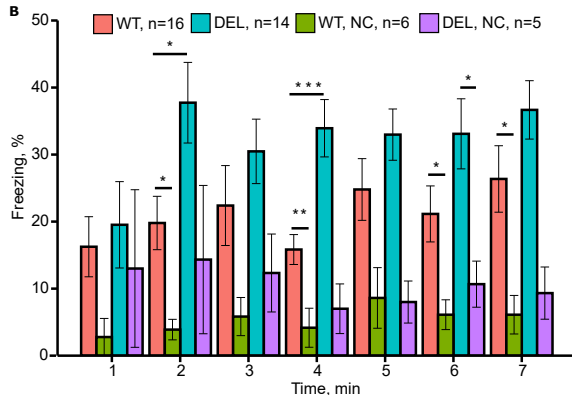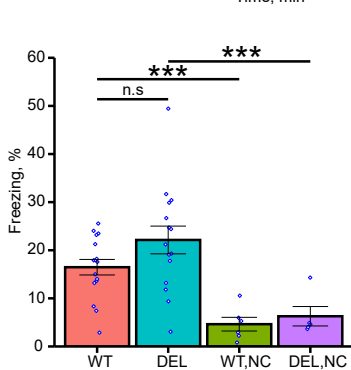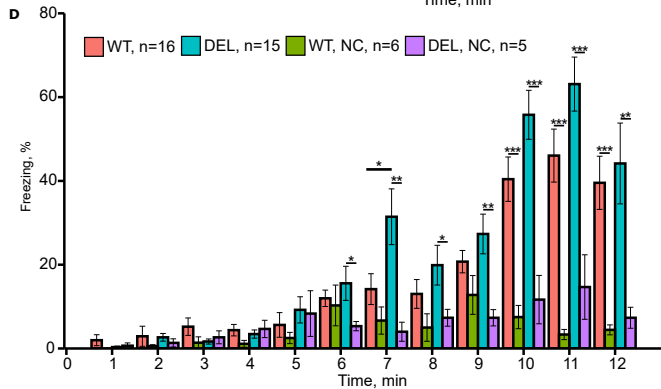

Supplement: Supplementary file 2 — Additional file 2. Fig. S2. Percentage freezing by minute during fear conditioning tests. [file 12915_2025_2138_MOESM2_ESM.pdf]

**A**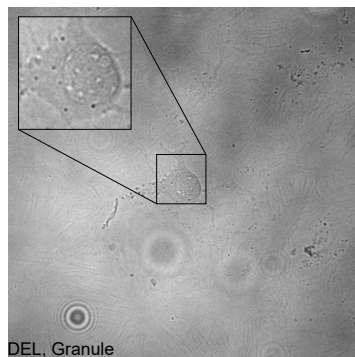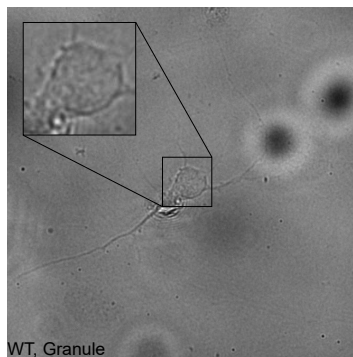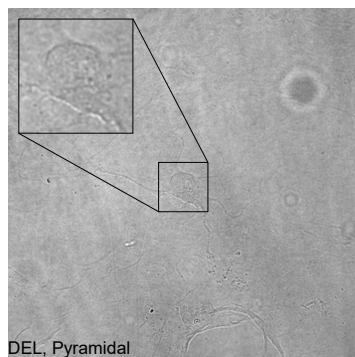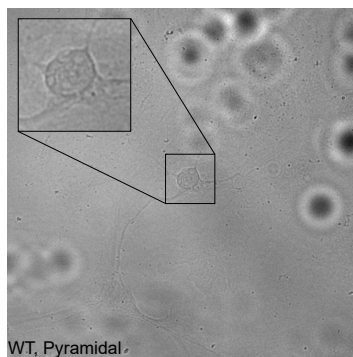**B**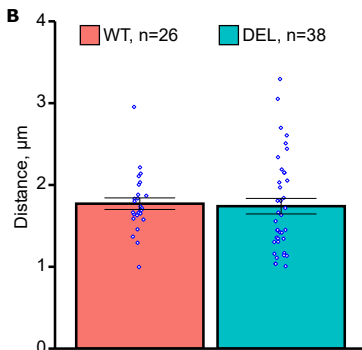**C**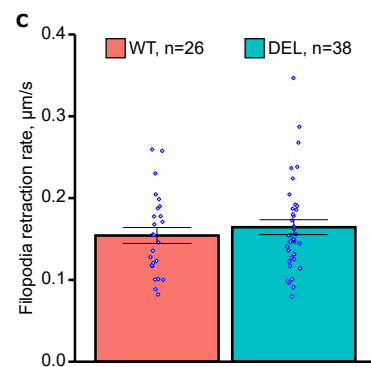**D**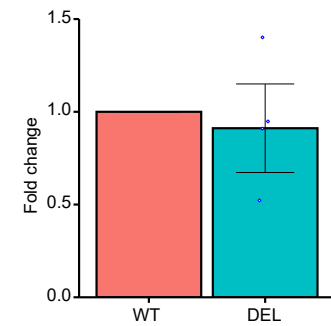

Ac-Tub

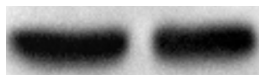

GAPDH

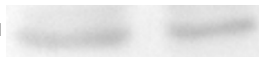**E**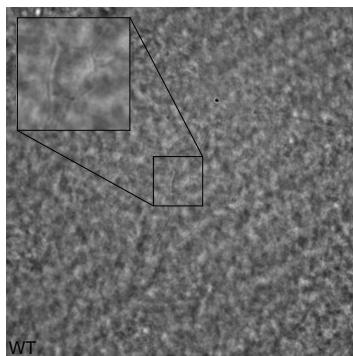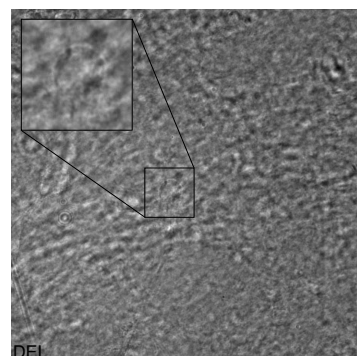

Supplement: Supplementary file 3 — Additional file 3. Fig. S3. Phase-contrast images and distance travelled by EB3-comets, filopodia retraction rate, and acetylated tubulin in hippocampal extracts. [file 12915_2025_2138_MOESM3_ESM.pdf]

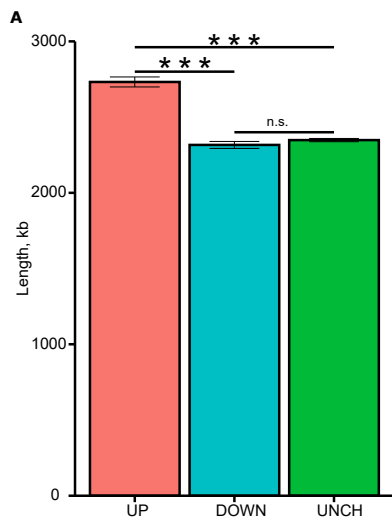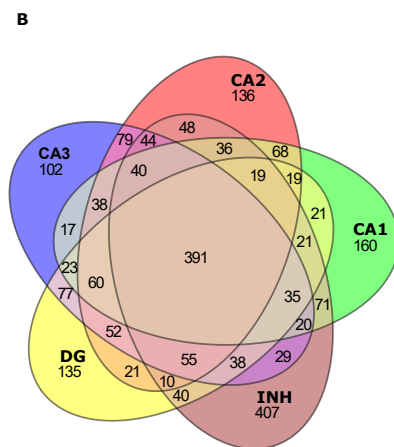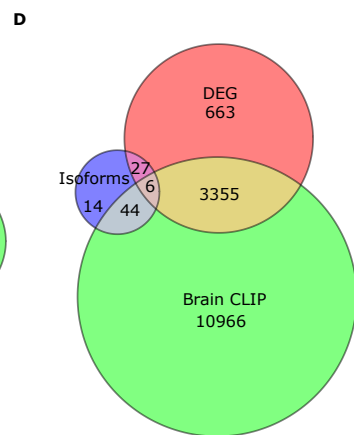

**C**

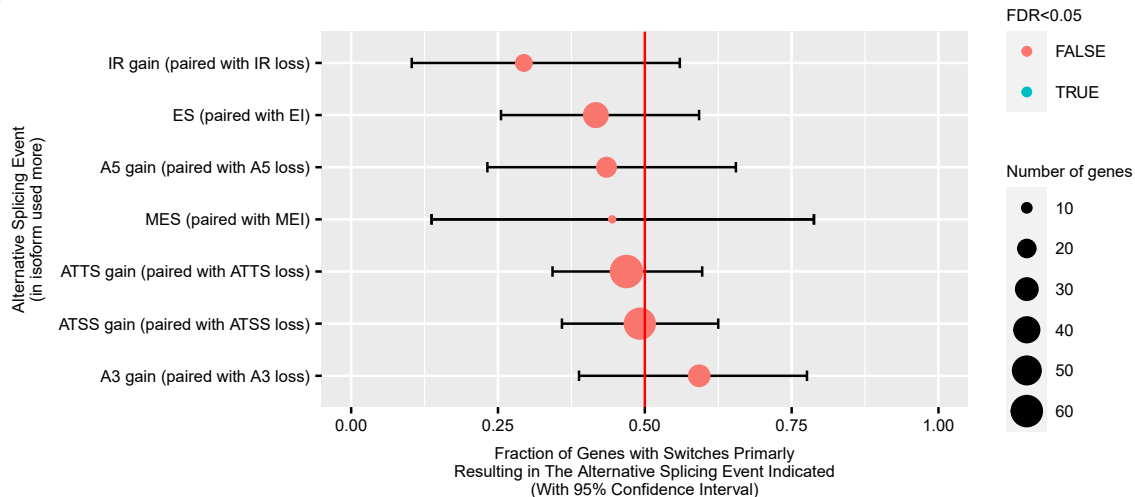

Supplement: Supplementary file 6 — Additional file 6. Fig. S4. Characteristics of the transcripts identified in RNA-seq. [file 12915_2025_2138_MOESM6_ESM.pdf]

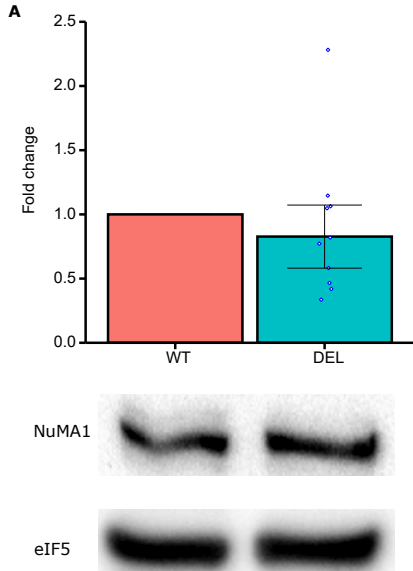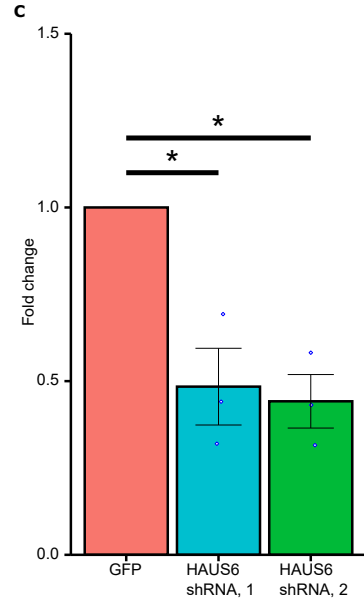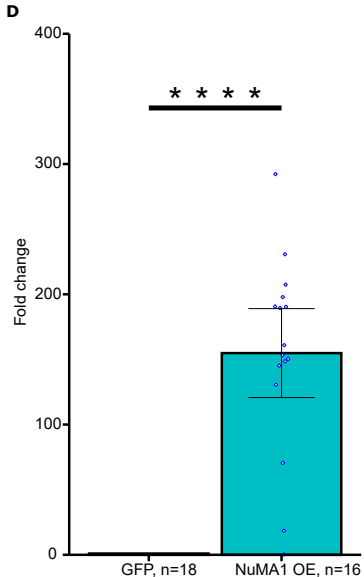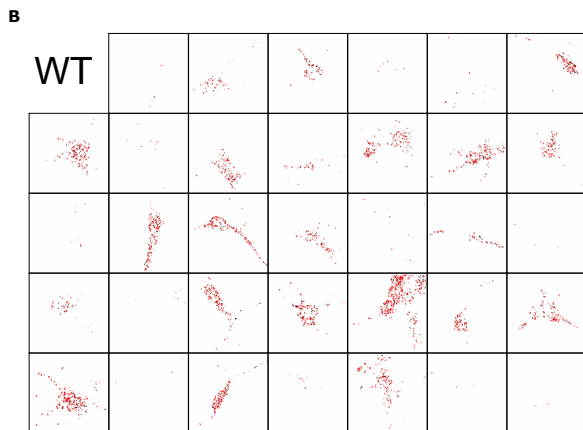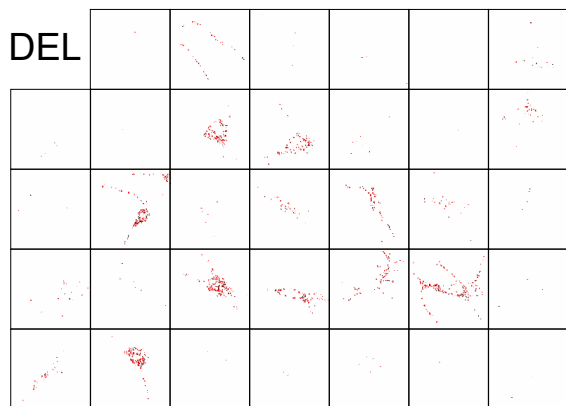

Supplement: Supplementary file 8 — Additional file 8. Fig. S5. NUMA1 expression and efficiency of NUMA1 over-expression and Haus6 knockdown. [file 12915_2025_2138_MOESM8_ESM.pdf]

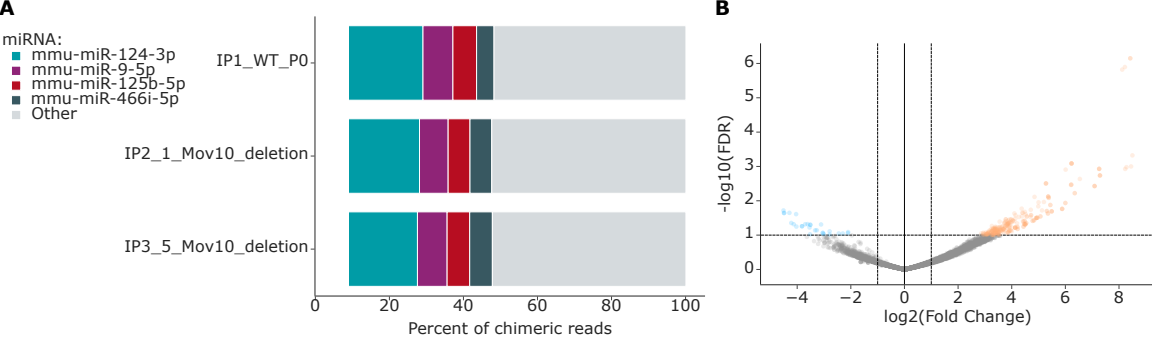

**C** DIV3 (3 days after nucleofection)

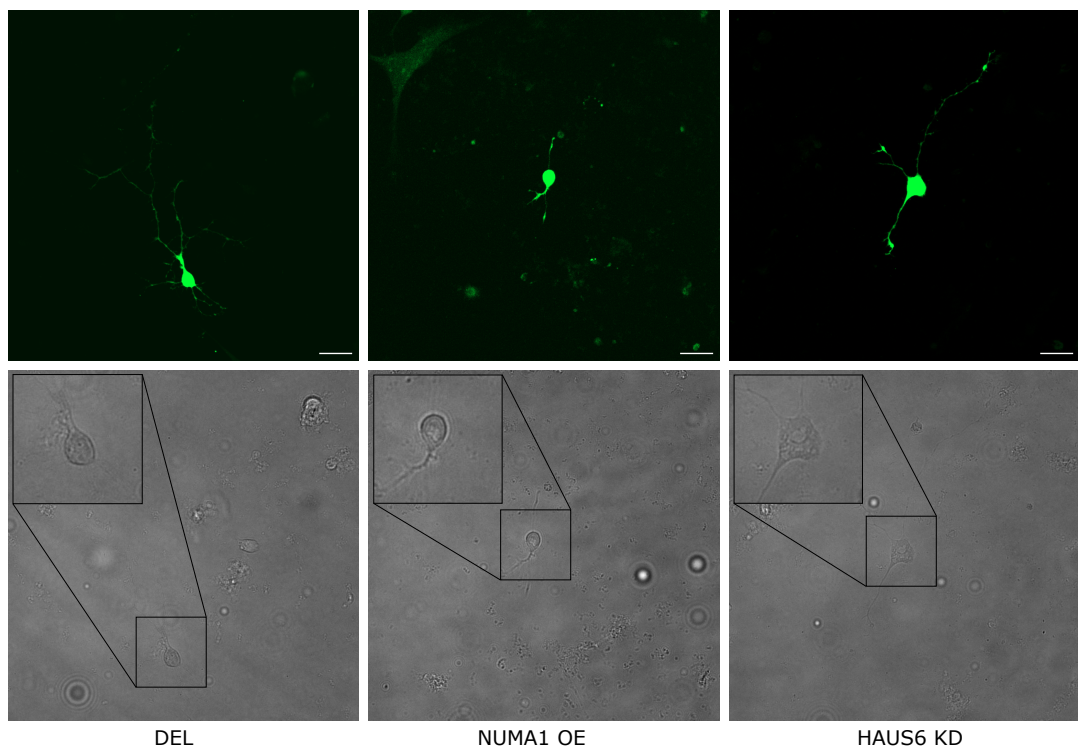

**D** DIV10 (3 days after lipofection)

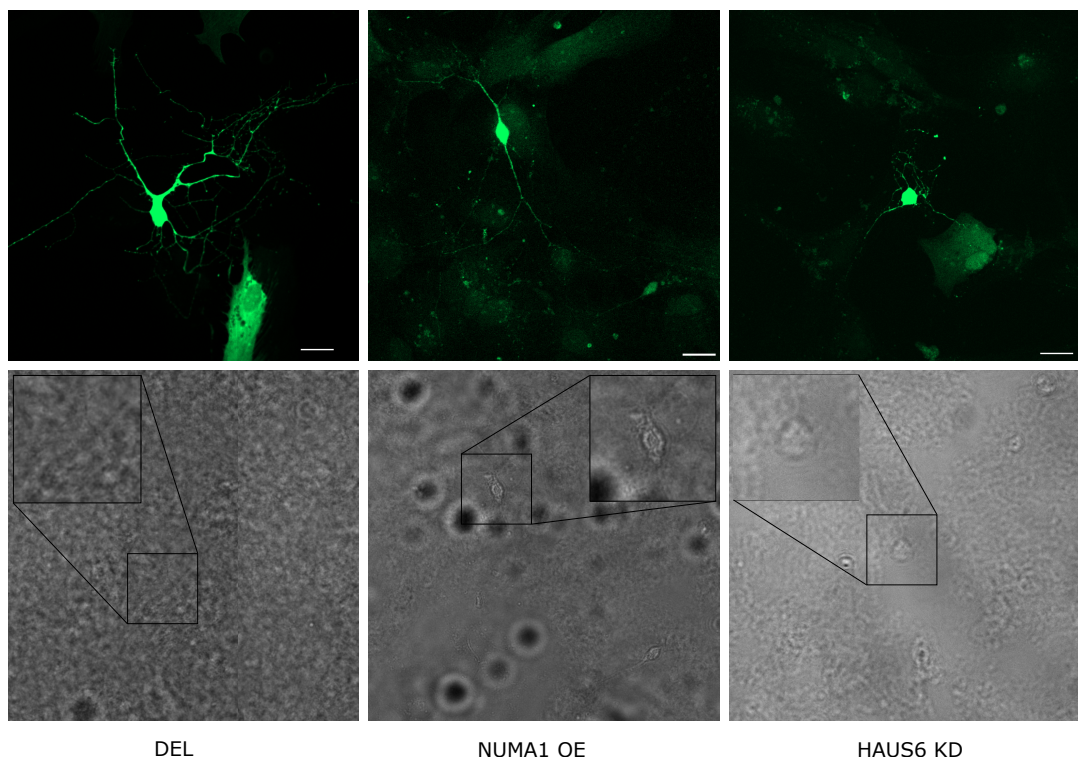

Supplement: Supplementary file 9 — Additional file 9. Fig. S6. miRNA and AGO2 peaks identified in eCLIP and representative neurons. [file 12915_2025_2138_MOESM9_ESM.pdf]
