# Supplementary material for: RNA helicase MOV10 suppresses fear memory and dendritic arborization and regulates microtubule dynamics in hippocampal neurons
Source: BMC Biol. 2025 Feb 6;23:36. doi: 10.1186/s12915-025-02138-6 (PMC11803958; doi:10.1186/s12915-025-02138-6)

**Creation of the *Mov10* Deletion mouse beginning with the *Mov10*^fl/fl^, removal of the neomycin cassette by FRT expression and crossing with the *Emx1*-Cre mouse**.

The MOV10 targeting construct shown in Figure 1 A, KO-first_condition_ready_118185_MGI:97054, was obtained from the European Mouse Mutant Cell Repository (EMMCR) described in Skarnes et al., 2011. The Genbank file for the targeting construct is available here:

<https://www.i-dcc.org/imits/targ_rep/alleles/43670/escell-clone-genbank-file>

To facilitate homologous recombination into the *Mov10* locus on chromosome 3, the construct included a 5’ homology region of 3.425 kb and a 3’ homology region of 6.2 kb to the *Mov10* locus. The plasmid was transfected into One-shot ccdB survival bacteria and selected on spectinomycin-containing LB plates (50 ug/ml). The small colonies contained the full-length plasmid, were picked and expanded and the isolated plasmid was verified by sequencing. The construct was then linearized using restriction enzyme AsiSI (NEB R0630S) and electroporated into C57BL/6 ES cells by the UIUC MCB Transgenic Mouse Facility (director Dr. Fuming Pan). His group performed neomycin selection to obtain 232 clones, which they individually frozen and extracted DNA from for screening by our lab. Clones that had a correctly targeted *Mov10* locus were identified by PCR screening with a 5’ primer set that included a forward primer from the genomic region upstream of the homology arm in intron 2 (primer Forward 5’330 below) and a reverse primer from the LacZ gene of the targeting construct (primer Reverse 5’330 below) to give a 4.6 kB product. A 3’ primer set was identified that was comprised of a forward primer from the neomycin gene in the targeting construct and a reverse primer downstream of the homology region in intron 18 to give a product of 8.55 kB (Forward 3’330 and Reverse 3’330, below).

**Primers**

Insertion of the 5’ homology arm of the targeting construct into chromosome 3

| Forward | 5’-330 | 5’ TAC TGC TCC CTG CCT CTT CCT ACT AAT 3’ |
| --- | --- | --- |
| Reverse | 5’-330 | 5’ GTA ATG GGA TAG GTC ACG TTG GTG TAG 3’ |

Jumpstart Accu-Taq, T_m_ 56°C. Product 4.6 kb

Insertion of the 3’ homology arm of the targeting construct into chromosome 3

| Forward | 3’-330 | 5’ CTA TCG CCT TCT TGA CGA GTT CTT CTG 3’ |
| --- | --- | --- |
| Reverse | 3’-330 | 5’ AGC TCT GAG GAC TAA GGG AGA AGT AAG 3’ |

Jumpstart Accu-Taq, T_m_ 56.5°C. Product 8.55 kb

10 clones were identified as being correctly targeted of which 6 were viable upon thawing. Clones 31, 33, 37, 39, and 41 were analyzed with the 3’ primer set and the 5’ primer shown below in a reverse image ethidium gel. Aberrantly targeted clones with only one homology arm are shown on the right.


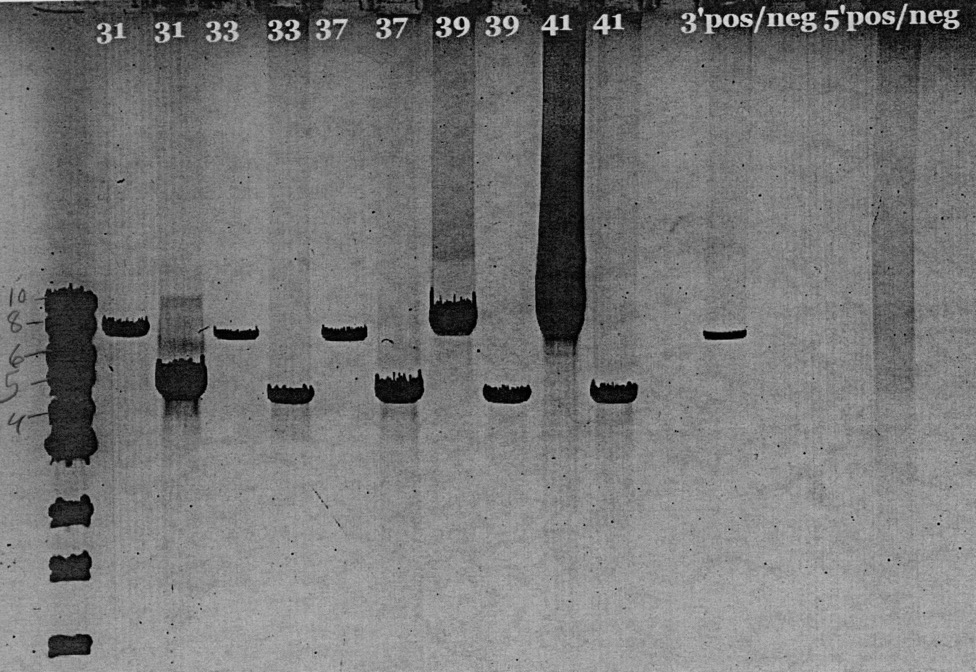


The clones were sequenced again to confirm the presence of the LoxP sites (clones 37, 41, 172 and 189) and two of the clones were introduced into albino C57BL/6 blastocysts for implantation into albino pseudopregnant females. Chimeric males were obtained and bred to albino females. Black pups were then screened for the presence of the targeted *Mov10* locus using the primers described below, which were designed to distinguish the targeted *Mov10* locus from the WT untargeted loci in a single reaction.

**tm1c. Target either intact intron 6, or the construct inserted into intron 6.**

| Forward | Intron-6-F | 5’ GAG TTT GAA GCC AGC CTG GGC TAT ATA A 3’ |
| --- | --- | --- |
| Reverse | Intron-6-R | 5’ GCC AGC ACT TAG CAG TGG AGA AG 3’ |
| Reverse | TCR | 5’ TTC GGA ATA GGA ACT TCG GGT CCG G 3’ |

| Intron-6-F + Intron-6-R | WT | 221 bp |
| --- | --- | --- |
| Intron-6-F + TCR | Floxed | 251 bp |


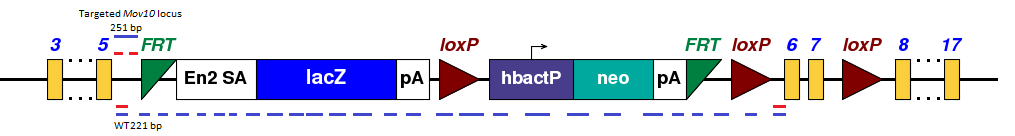


Red primers shown on top are to the targeted locus (250 bp product) and the bottom primer set is to the WT *Mov10* locus (intron 6) (221 bp product).


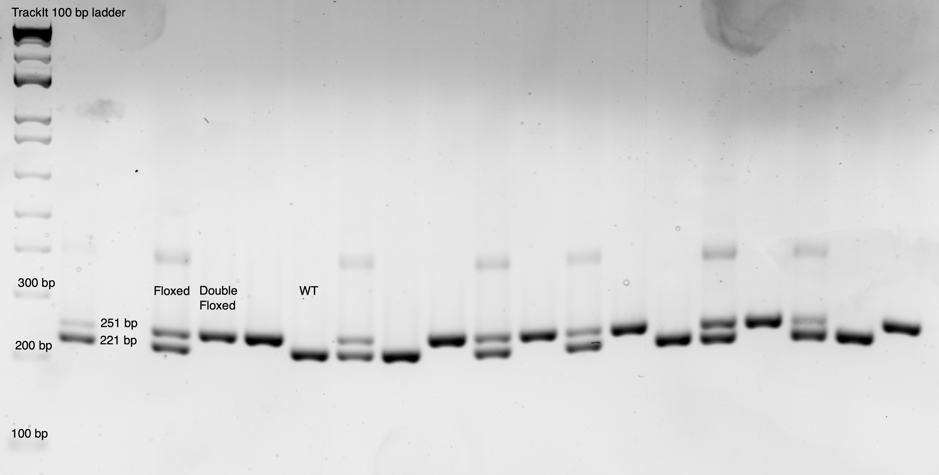


The inverted image of ethidium bromide gel shows results of PCR screening of pups from heterozygous crosses. Lane 1- Marker ladder with sizes indicated. “Floxed” denotes mouse with WT *Mov10* and targeted (floxed) *Mov10* allele; WT mice inherited only the WT *Mov10* allele. Several mice in this experiment inherited two targeted *Mov10* alleles, “Double floxed”.

After we created the *Mov10^fl/fl^* mouse, we crossed it with a transgenic mouse expressing the FRT recombinase <https://www.jax.org/strain/009086> to remove the neomycin cassette because of its reported effect on flanking DNA (Xue et al., 2015).

Offspring mice were screened for the absence of neomycin using primers below

| Forward | Neo1F-712 | 5’ CTT TTC TGG ATT CAT CGA CTG TG 3’ |
| --- | --- | --- |
| Reverse | Neo1R-712 | 5’ GCT CAG AAG AAC TCG TCA AGA AG 3’ |

T_m_ 60°C, Product size 192 bp.

We then crossed the resulting floxed mouse without neomycin to the *Emx1*-Cre recombinase mouse [https://www.jax.org/strain/005628](https://urldefense.proofpoint.com/v2/url?u=https-3A__www.jax.org_strain_005628&d=DQMFAg&c=8hUWFZcy2Z-Za5rBPlktOQ&r=cJadr89QjOo1nLiX0fqKiwymKQo2Mvs8651NVg5F7GY&m=fzQYAkc1vdCg-kI6cAQb4iGDE1GdqaE5mUqiK41vWT0&s=pQg4KYlYhtxNyCTkMIuX5dHzA9izLc0BHWE6A8Oopyw&e=) to disrupt MOV10 expression in forebrain neurons. We used the primers below to evaluate the presence or absence of Cre recombinase on chromosome 6.

| Forward | oIMR1-1084 | 5’ GCG GTC TGG CAG TAA AAA CTA TC 3’ |
| --- | --- | --- |
| Reverse | oIMR1-1085 | 5’ GTG AAA CAG CAT TGC TGT CAC TT 3’ |
| Forward | oIMR1-4170 | 5’ AAG GTG TGG TTC CAG AAT CG 3’ |
| Reverse | oIMR1-4171 | 5’ CTC TCC ACC AGA AGG CTG AC 3’ |

| oIMR1-1084 + oIMR1-1085 | Mutant | 102 bp |
| --- | --- | --- |
| oIMR1-4170 + oIMR1-4171 | WT | 378 bp |

T_m_ 65-68°C, 0.5°C per cycle for 10 cycles, then 60°C for 18 cycles.


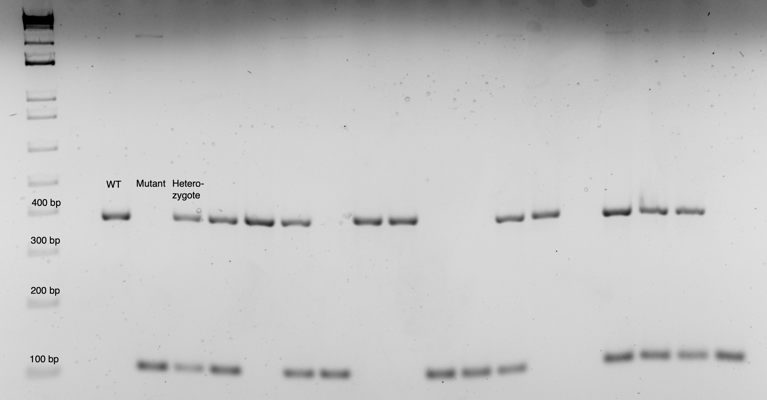


***Mov10* HET.** Please note that this mouse has been described in an earlier publication Skariah et al., 2017 but the primers will be reproduced here for completeness.

Reverse VP76-R primer targets the insertion construct that disrupts the allele.

| Forward | mMov10-F | 5’ GCG ATG CCT AGC AAG TTC AG 3’ |
| --- | --- | --- |
| Reverse | mMov10-R-250 | 5’ CCC AGC GGT CTA GTT TGA AG 3’ |
| Reverse | VP76-R | 5’ CCA ATA AAC CCT CTT GCA GTT GC 3’ |

| mMov10-F + mMov10-R-250 | WT | 250 bp |
| --- | --- | --- |
| mMov10-F + VP76-R | HET | 229 bp |

T_m_ 60°C.


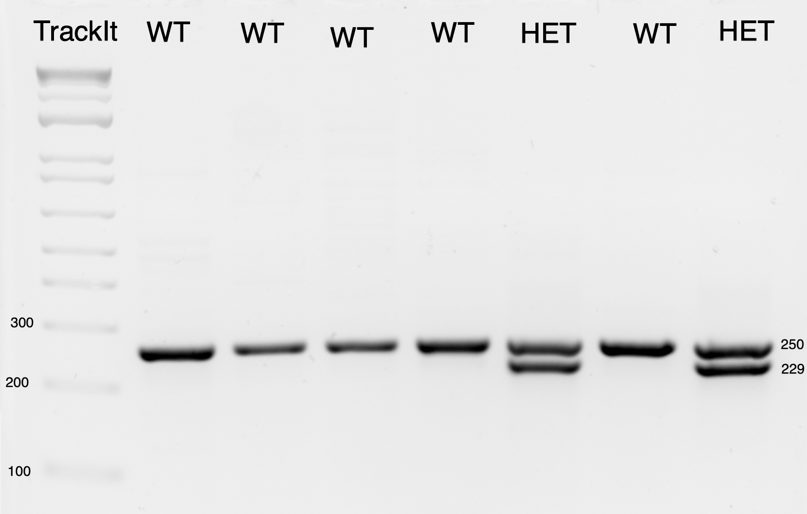


**tm1d. Target the junction after deletion of exon 6 and 7 of *Mov10* through FloxP/Cre.**

| Forward | tm1c_F | 5’ AAG GCG CAT AAC GAT ACC AC 3’ |
| --- | --- | --- |
| Reverse | Floxed_LR | 5’ ACT GAT GGC GAG CTC AGA CC 3’ |

| tm1c_F + Floxed_LR | Deletion | 174 bp |
| --- | --- | --- |

T_m_ 58°C.


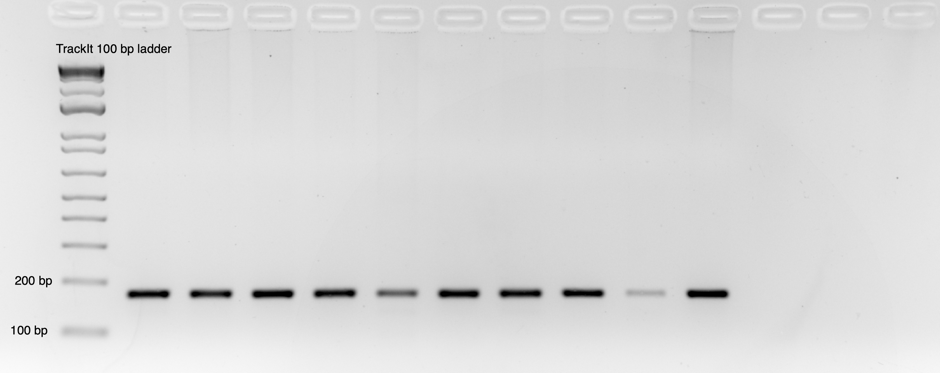

Supplement: Supplementary file 10 — Additional file 10. Creation of the Mov10 Deletion mouse. [file 12915_2025_2138_MOESM10_ESM.docx]
