# Supplementary material for: RNA helicase MOV10 suppresses fear memory and dendritic arborization and regulates microtubule dynamics in hippocampal neurons
Source: BMC Biol. 2025 Feb 6;23:36. doi: 10.1186/s12915-025-02138-6 (PMC11803958; doi:10.1186/s12915-025-02138-6)
Supplement: Supplementary file 11 — Additional file 11. Original uncropped Blots for Fig. 1B, Fig. S3D, and Fig. S5A. [file 12915_2025_2138_MOESM11_ESM.pdf]

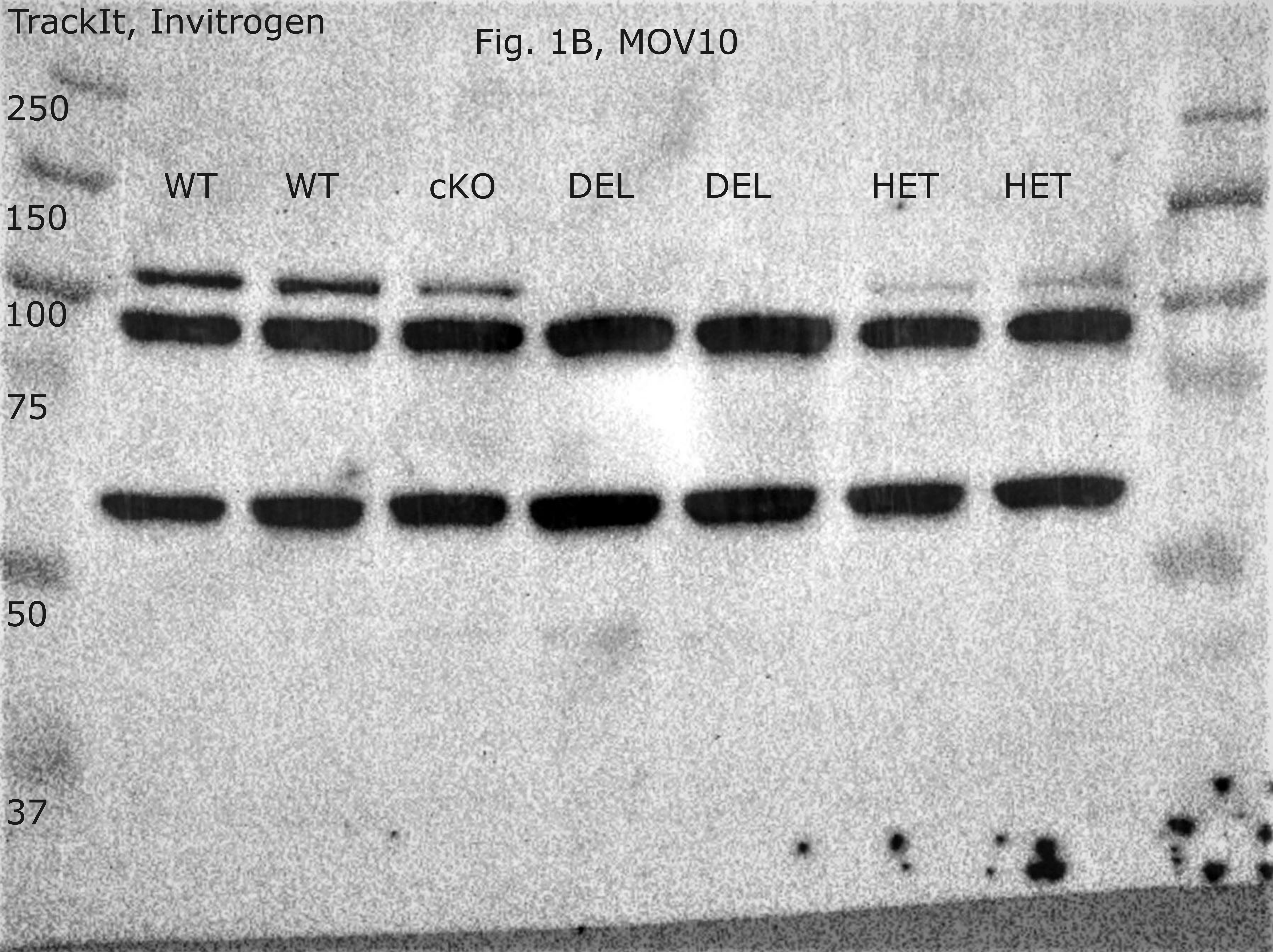
TrackIt, Invitrogen
Fig. 1B, MOV10
250
150
100
75
50
37
WT
WT
cKO
DEL
DEL
HET
HET

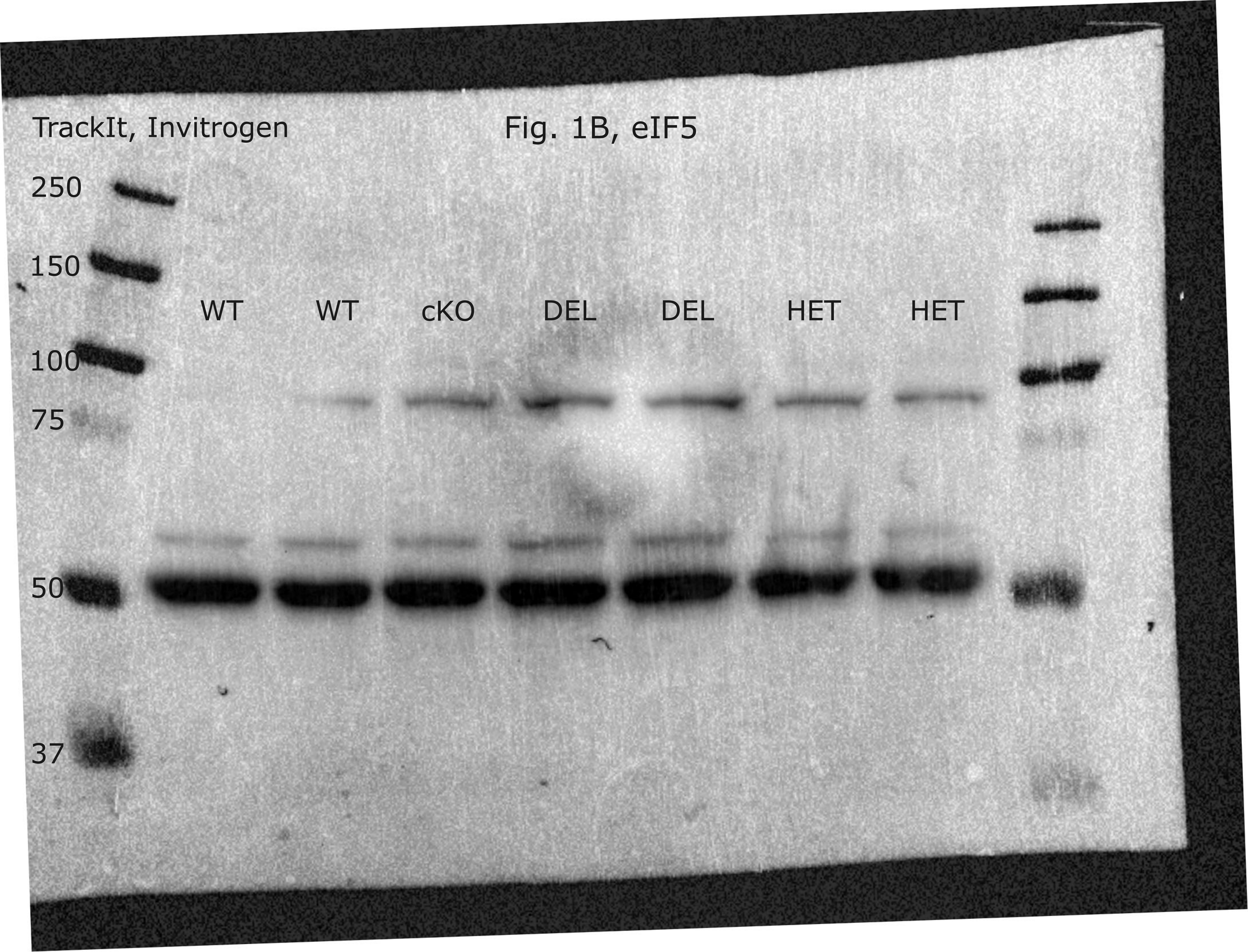
TrackIt, Invitrogen
Fig. 1B, eIF5
250
150
100
75
50
37
WT
WT
cKO
DEL
DEL
HET
HET

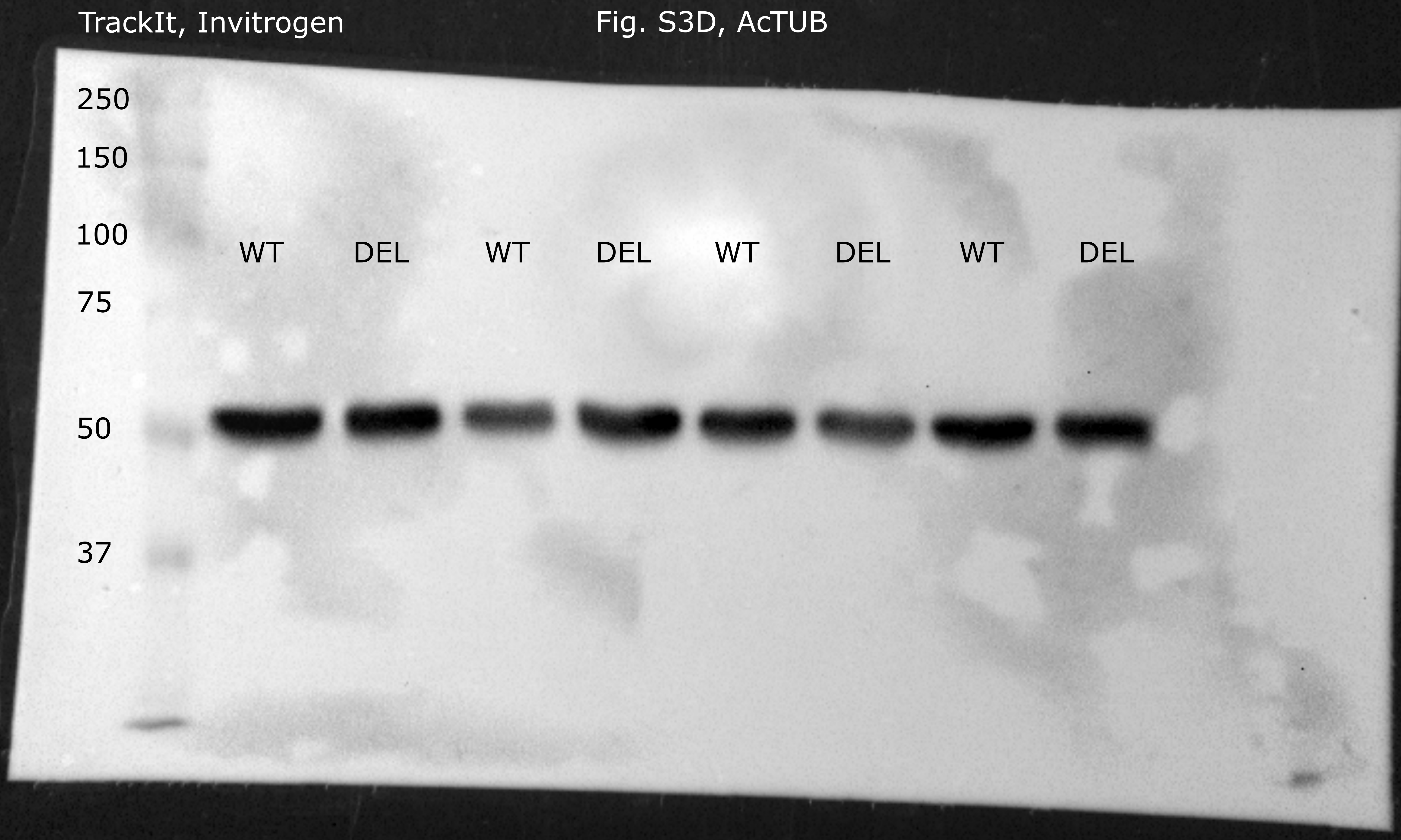
TrackIt, Invitrogen
Fig. S3D, AcTUB
250
150
100
75
50
37
WT
DEL
WT
DEL
WT
DEL
WT
DEL

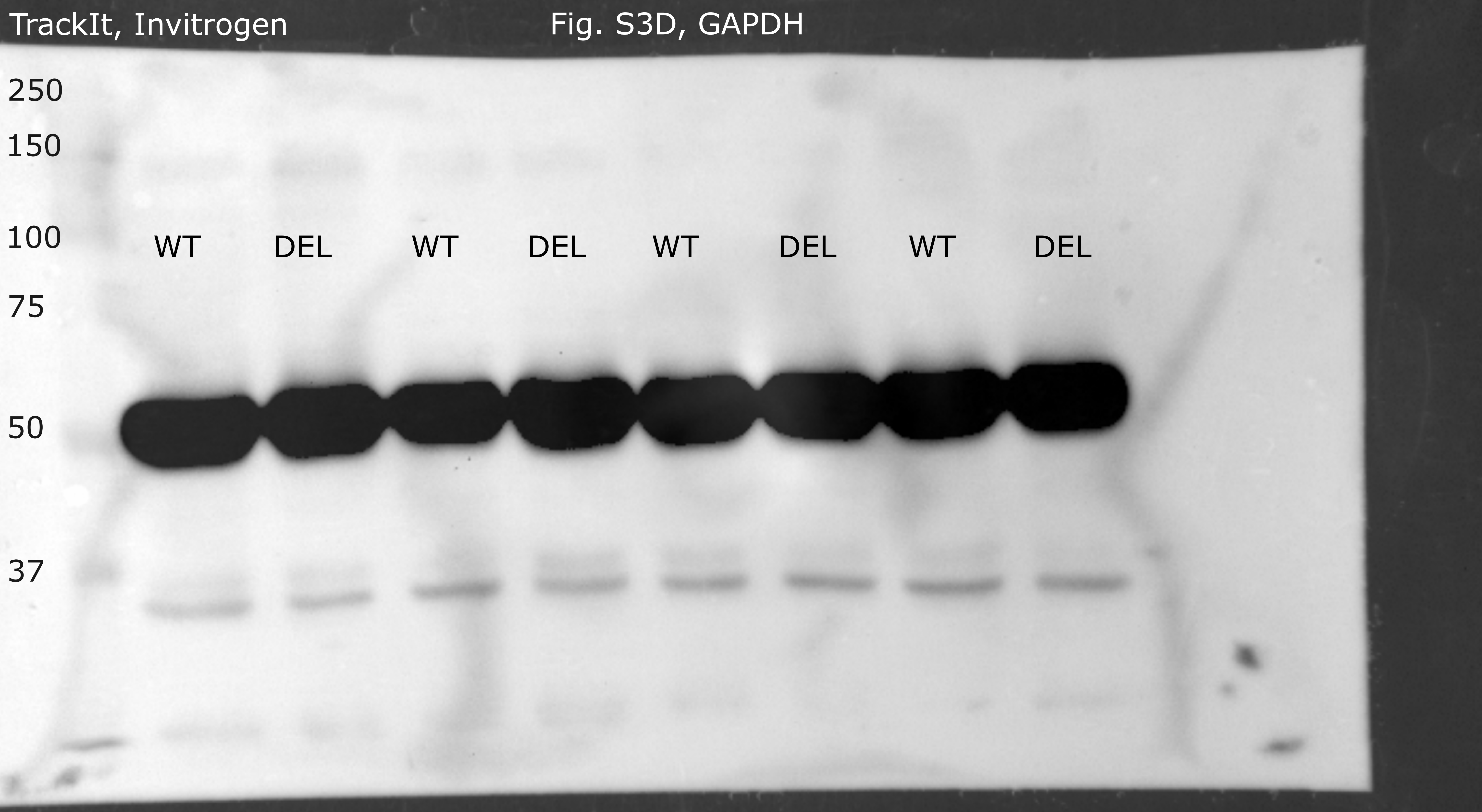
TrackIt, Invitrogen
Fig. S3D, GAPDH
250
150
100
75
50
37
WT
DEL
WT
DEL
WT
DEL
WT
DEL

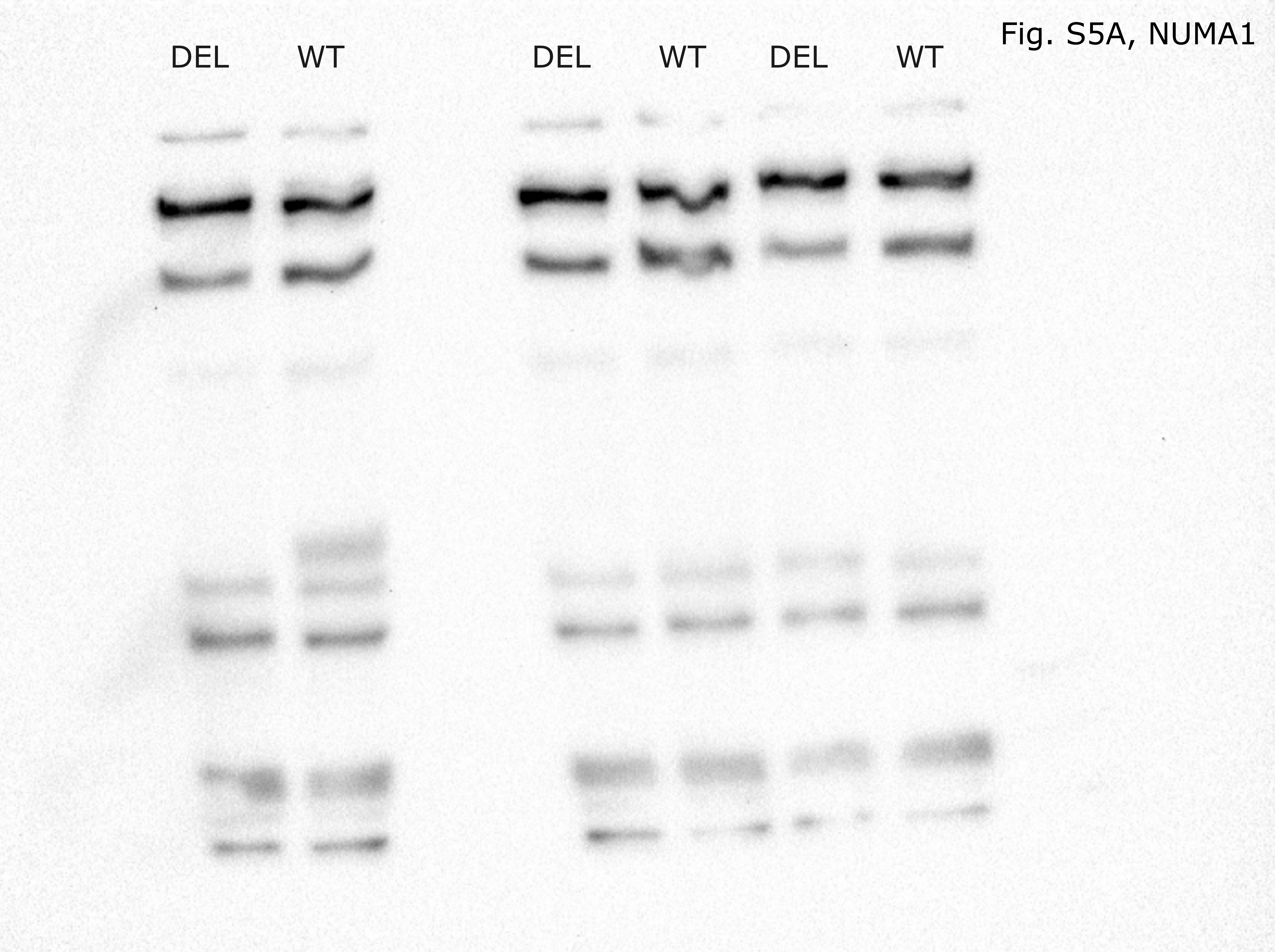

Fig. S5A, NUMA1
DEL
WT
DEL
WT
DEL
WT

Fig. S5A, eIF5

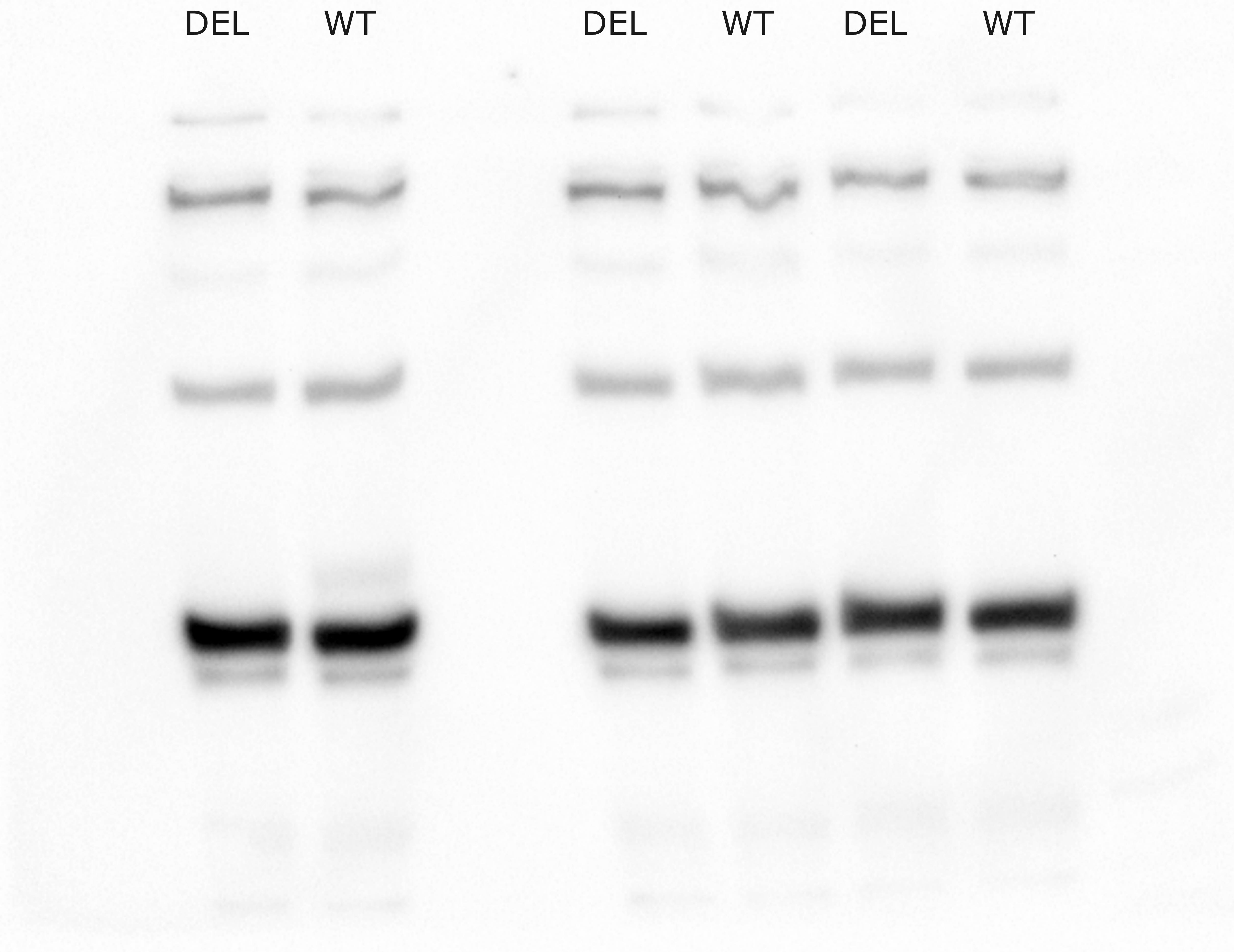

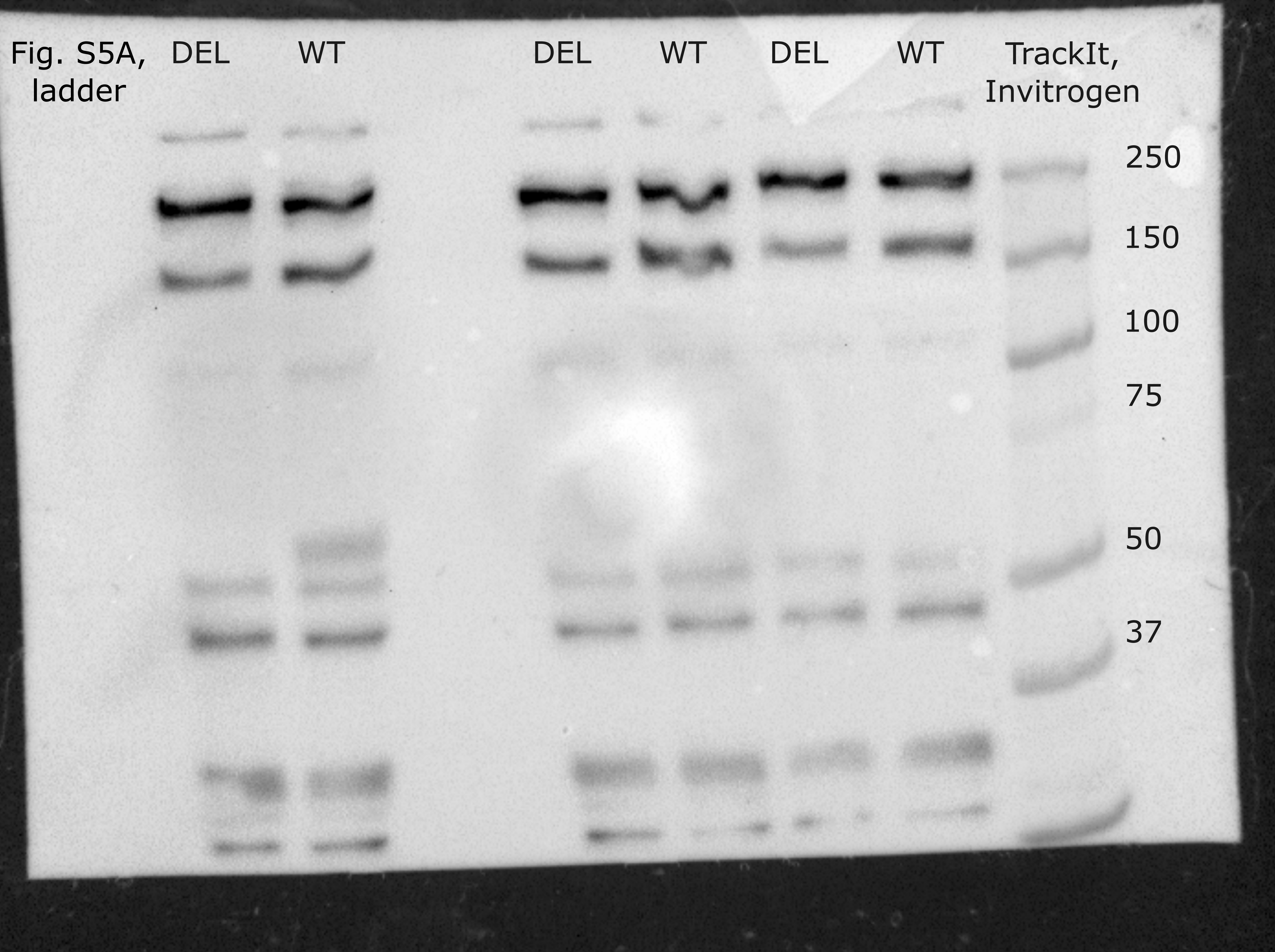
Fig. S5A, ladder
DEL
WT
DEL
WT
DEL
WT
TrackIt, Invitrogen
250
150
100
75
50
37
